# Secondary metabolite profiling of *Pseudomonas aeruginosa* isolates reveals rare genomic traits

**DOI:** 10.1128/msystems.00339-24

**Published:** 2024-04-15

**Authors:** Rachel L. Neve, Emily Giedraitis, Madeline S. Akbari, Shirli Cohen, Vanessa V. Phelan

**Affiliations:** 1 Department of Immunology and Microbiology, School of Medicine, University of Colorado Anschutz Medical Campus, Aurora, Colorado, USA; 2 Department of Pharmaceutical Sciences, Skaggs School of Pharmacy and Pharmaceutical Sciences, University of Colorado Anschutz Medical Campus, Aurora, Colorado, USA; MS Bioscience, Maringá, Brazil

**Keywords:** *Pseudomonas aeruginosa*, metabolomics, secondary metabolites

## Abstract

*Pseudomonas aeruginosa* is a ubiquitous Gram-negative opportunistic pathogen with remarkable phylogenetic and phenotypic variabilities. In this work, we applied classical molecular networking analysis to secondary metabolite profiling data from seven *Pseudomonas aeruginosa* strains, including five clinical isolates from the lung secretions of people with cystic fibrosis (CF). We provide three vignettes illustrating how secondary metabolite profiling aids in the identification of rare genomics traits in *P. aeruginosa*. First, we describe the identification of a previously unreported class of acyl putrescines produced by isolate mFLRO1. Secondary analysis of publicly available metabolomics data revealed that acyl putrescines are produced by <5% of *P. aeruginosa* strains. Second, we show that isolate SH3A does not produce di-rhamnolipids. Whole-genome sequencing and comparative genomics revealed that SH3A cannot produce di-rhamnolipids because its genome belongs to clade 5 of the *P. aeruginosa* phylogenetic tree. Previous phylogenetic analysis of thousands of *P. aeruginosa* strains concluded that <1% of publicly available genome sequences contribute to this clade. Last, we show that isolate SH1B does not produce the phenazine pyocyanin or rhamnolipids because it has a one-base insertion frameshift mutation (678insC) in the gene *rhlR*, which disrupts *rhl*-driven quorum sensing. Secondary analysis of the tens of thousands of publicly available genomes in the National Center for Biotechnology Information (NCBI) and the *Pseudomonas* Genome Database revealed that this mutation was present in only four *P. aeruginosa* genomes. Taken together, this study highlights that secondary metabolite profiling combined with genomic analysis can identify rare genetic traits of *P. aeruginosa* isolates.

**IMPORTANCE:**

Secondary metabolite profiling of five *Pseudomonas aeruginosa* isolates from cystic fibrosis sputum captured three traits present in <1%–5% of publicly available data, pointing to how our current library of *P. aeruginosa* strains may not represent the diversity within this species or the genetic variance that occurs in the CF lung.

## INTRODUCTION

*Pseudomonas aeruginosa* is a ubiquitous Gram-negative bacterium responsible for both acute and chronic infections. The remarkable phylogenetic and phenotypic diversity of *P. aeruginosa*, including varied virulence mechanisms, conversion to mucoid by overproduction of alginate, and altered quorum sensing (QS) pathways contribute to its survival in varied environments (1–5). One mechanism *P. aeruginosa* uses to interact with its environment is the production of secondary metabolites (6–8). The secondary metabolome of *P. aeruginosa* has been extensively characterized and consists of a small number of molecular families, including homoserine lactones (HSLs), phenazines (PHZs), alkyl quinolones (AQs), rhamnolipids (RLs), and the siderophores pyochelin (PCH) and pyoverdines (PVDs) (7, 9–12). These compounds are known to mediate a variety of virulence mechanisms used by *P. aeruginosa* in the host environment, including swarming motility, QS, iron acquisition, and the production of reactive oxygen species (10, 11, 13). Although PVD is required for acute infections, its production is often lost over the course of chronic infection (14, 15).

The production of secondary metabolites by *P. aeruginosa* is dependent, in part, on the functionality of their biosynthetic gene clusters (BGCs), the QS pathways that regulate their production, and environmental iron concentration (16). The BGCs of secondary metabolites are well conserved in the genomes of *P. aeruginosa* isolates. Despite distinct biosynthetic pathways, the biosynthesis of PHZs, AQs, PCH, and PVDs is dependent upon the availability of exogenous aromatic amino acids or the biosynthesis of chorismate, a shared precursor for aromatic amino acid and secondary metabolite biosynthesis (17–20). Chorismate is required for the biosynthesis of the PHZs, AQs, and PCH, while tyrosine is required for the production of PVDs. Notably, expression of genes for aromatic compound degradation and genes for secondary metabolite biosynthesis is also necessary for full virulence of *P. aeruginosa* (21).

The QS system of *P. aeruginosa* that regulates secondary metabolite biosynthesis is composed of three interdependent regulatory circuits: *las*, *rhl*, and *pqs* systems (22). Historically considered hierarchical, the three QS systems are inherently intertwined with overlapping regulons. In the *las* QS system, LasI produces the autoinducer 3-oxo-dodecanoyl-L-homoserine lactone (3-oxo-C12-HSL), which is subsequently bound by the LasR transcriptional regulator (23, 24). LasR bound to 3-oxo-C12-HSL dimerizes and activates a variety of transcriptional pathways, including the *rhl* QS system. In *rhl* QS, RhlI produces *N*-butanoyl-L-homoserine lactone (C4-HSL), which binds to the RhlR transcriptional regulator, enabling dimerization and activation of its regulon (25). The RhlI/RhlR system directly controls RL production (26). Both *las* and *rhl* QS systems regulate the *pqs* QS system through the transcriptional regulator PqsR (MvfR) (22). PqsR regulates the production of AQs, including the autoinducers 2-heptyl-4-quinolone (HHQ) and *Pseudomonas* quinolone signal (PQS), with PQS as a more potent inducer of PqsR than HHQ (22). The BGC for the AQs includes the protein PqsE, which is not required for AQ production but is required for production of the PHZs, including pyocyanin (PYO) (25). The function of PqsE in QS has not been fully elucidated, but it is known to function as a chaperone providing stability to RhlR and may also produce an uncharacterized signaling molecule (27, 28). Both RhlR and PqsE are required for production of PYO by *P. aeruginosa* (29). Mutations within the QS pathways are common. Mutations affecting LasR are more common than RhlR (30). However, some isolates with LasR QS mutations are still capable of producing secondary metabolites due to secondary mutations within other transcriptional regulators, including MexT, which can activate the *rhl* QS pathways (31, 32).

Secondary metabolite production by *P. aeruginosa* is also regulated by iron concentration (33). Under iron replete, aerobic conditions, soluble ferrous iron reacts with oxygen to produce insoluble ferric iron. In response, *P. aeruginosa* increases production of PHZs to reduce the ferric iron to ferrous iron (34). After transport into the cell, intracellular ferrous iron binds to the ferric uptake regulator (Fur) protein, which represses siderophore and rhamnolipid biosynthesis (35, 36). Under iron deplete conditions, *P. aeruginosa* produces the siderophores PCH and PVDs to acquire iron from the environment, and the Fur-regulated PrrF non-coding small RNAs repress anthranilate degradation, promoting the production of PQS (35, 37, 38).

Recently, untargeted liquid chromatography tandem mass spectrometry (LC-MS/MS) profiling of the *P. aeruginosa* secondary metabolome has been applied to identify markers of virulence, measure the effect of nutritional changes on QS pathways, and capture the diversity of the metabolites produced (7, 8, 10, 39, 40). Targeted LC-MS/MS profiling of HSL and AQ metabolites was used to unambiguously determine the functionality of *las* QS signaling of 176 environmental isolates, illustrating that LasR-defective isolates are ubiquitous (32). We hypothesized that under identical growth conditions, secondary metabolite profiling would provide a method for capturing alterations in metabolite biosynthesis and/or QS due to mutations in the genomes of *P. aeruginosa* isolates.

As proof of principle, we applied untargeted LC-MS/MS secondary metabolite profiling to five isolates of *P. aeruginosa* from cystic fibrosis (CF) sputum. CF is a genetic disease characterized by chronic and recurrent airway infections caused by *P. aeruginosa* (41). The isolates nmFLRO1, mFLRO1, SH1B, SH2D, and SH3A and laboratory strains PAO1 and PA14 were cultured in synthetic cystic fibrosis medium 2 (SCFM2), a chemically defined medium complemented with mucin and DNA developed to mimic the nutritional environment of the CF lung (42–44). Using classical molecular networking, we evaluated the metabolomics data for metabolites only produced or not produced by single isolates (45). Using this approach, we discovered that mFLRO1 produced a previously unreported class of acyl putrescines from the conjugation of putrescine with various fatty acids, SH3A only produces mono-RLs due to its genome similarity to phylogenetic clade 5 representative strain PA-W1, and SH1B has a one-base frameshift mutation in *rhlR* that disrupts QS signaling. Secondary analysis of publicly available secondary metabolite profiling and/or genomic data indicated that acyl putrescines are produced by ~5%; clade 5 genomes represent <1%; and the *rhlR* frameshift mutation occurs in ~0.0004% of the *P. aeruginosa* population. These results illustrate that secondary metabolite profiling combined with secondary analysis of publicly available data can be used to identify rare genomic variants that impact QS and metabolite biosynthesis in *P. aeruginosa* isolates.

## RESULTS AND DISCUSSION

**Fig 1:**
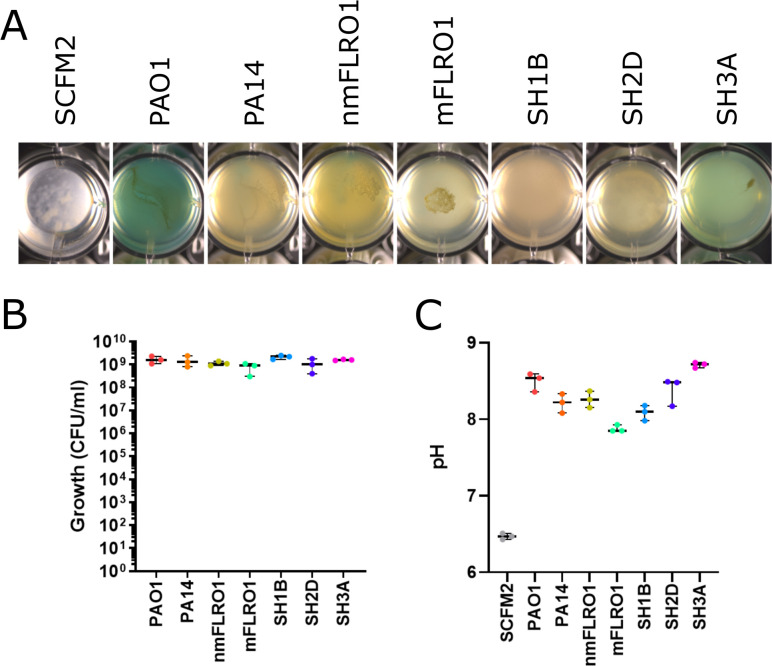
Growth of seven *P. aeruginosa* strains in SCFM2. (**A**) Representative phenotypes of strains grown statically in 1 mL of SCFM2 (*n* = 3) in a 48-well plate for 48 h at 37°C. Photographs of replicate growth wells are shown in Fig. S1 in the supplemental material. (**B**) Quantified growth of each strain as colony-forming units (CFU) per milliliter. (**C**) pH after mechanical disruption of cultures.

Five *P. aeruginosa* de-identified CF clinical isolates, including nmFLRO1, mFLRO1, SH1B, SH2D, and SH3A, were selected and subjected to secondary metabolite profiling (Table S1). No specific criteria were used to select the isolates for analysis. Isolates nmFLRO1 and mFLRO1 were isolated at the University of California, San Diego Adult CF Clinic, and previously used to represent non-mucoid (nmFLRO1) and mucoid (mFLRO1) phenotypes in model cultures and volatile metabolite analysis (44, 46, 47). Strains SH1B, SH2D, and SH3A were isolated from an individual with CF in Hanover, Germany, before (10/92, SH1B), during (8/93, SH2D), and after (9/95, SH3A) a transient infection with *Burkholderia cenocepacia* H111 (43). The isolates were cultured in SCFM2 alongside laboratory strains PAO1 and PA14, and gross morphology of the cultures was visualized using a stereo microscope (Fig. 1A; Fig. S1).

All *P. aeruginosa* strains formed macrostructures at the air-liquid interface of the cultures, which descended into aggregates on the bottom of the wells. Aligning with our previous results, PAO1 cultures in SCFM2 were blue, indicating production of the phenazine PYO (7). The cultures of the other strains were less visually distinct, ranging from moderate yellow (nmFLRO1) to faint green (SH3A) and colorless (mFLRO1). The color of the cultures and the complexity of the macrostructures were strain dependent and somewhat variable between replicates (Fig. S1). Although most of the macroaggregate structures were dispersed across the top of the well, two replicates of mFLRO1 formed a tighter pattern. The variability in culture color and aggregation between the strains was not due to differences in growth, as there was similar recovery of colony-forming units (CFU) (CFU per milliliter) after disruption of the aggregates from all cultures (Fig. 1B). Although all cultures became alkaline compared to media control, those of mFLRO1 were less basic than the other strains (Fig. 1C).

### Classical molecular networking analysis of isolates cultured in SCFM2

To determine whether the CF isolates produced the same suite of secondary metabolites as the laboratory strains, we subjected culture extracts to LC-MS/MS metabolomics and classical molecular networking (CMN) using the Global Natural Products Social Molecular Networking (GNPS) analysis environment (45). CMN organizes structurally related metabolites into molecular families by leveraging the concept that molecules with similar chemical structures have similar fragmentation patterns in their tandem mass spectrometry (MS/MS) spectra. In the molecular network, the MS/MS spectra of molecular ions are represented as nodes, and the relatedness between the MS/MS spectra is illustrated by the thickness of the connecting edges. Each node in a classical molecular network is most often composed of MS/MS spectra from multiple samples. Metadata groups denoting strain name (“strain,” e.g., “PAO1”) and type (“origin,” e.g., “lab”) were used to identify which samples contributed MS/MS spectra to each node. Simultaneously, the experimental MS/MS spectra were searched against the publicly available MS/MS libraries within GNPS, providing putative annotation of compounds. Putative annotations were verified by comparing experimental data to authentic commercial standards and/or published data (48, 49). The number of nodes shared by each combination of sample groups was quantified.

**Fig 2:**
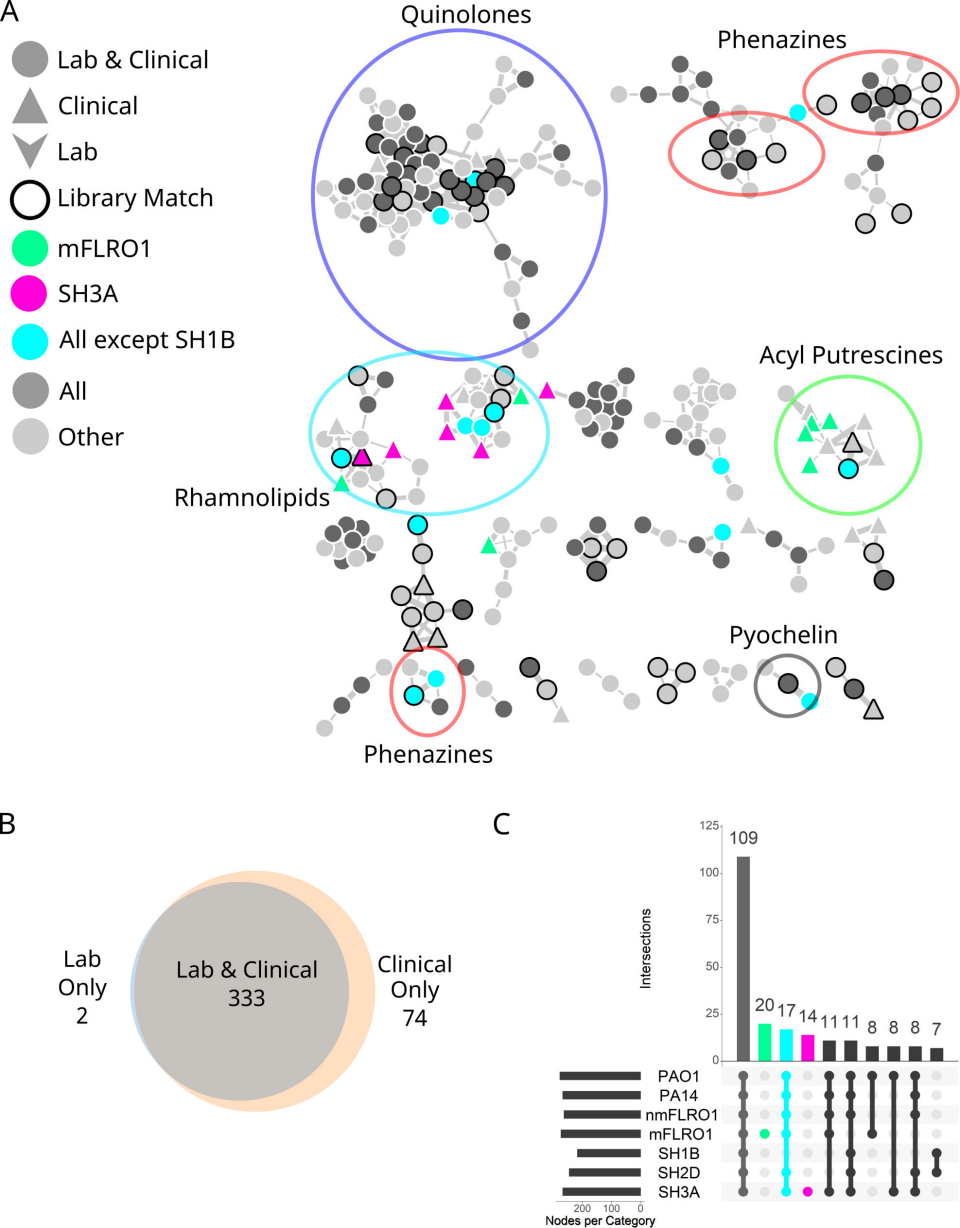
Molecular ions detected from the cultures of seven *P. aeruginosa* strains. (**A**) Classical molecular network of seven *P. aeruginosa* strains cultured in SCFM2. Nodes corresponding to the five molecular families discussed in the main text are highlighted by large ovals. Nodes with ≤1 connection to another node were omitted for visual clarity. The full molecular network is in Fig. S2 in the supplemental material. Nodes represent molecular ions (precursor mass and MS/MS spectra). The widths of the lines connecting nodes (edges) represent the similarity of the MS/MS fragmentation of the connected nodes. Nodes with black outlines indicate a spectral match between the data and MS/MS spectra within the GNPS spectral libraries. Node shape represents whether the molecular ion was detected from cultures of clinical isolates (triangle) or laboratory strains and clinical isolates (circle). Node color indicates whether the molecular ion was detected in cultures from all strains (dark gray), strain mFLRO1 (green), strain SH3A (pink), all strains except SH1B (teal) or combinations of strains (light gray) to highlight the strain-specific analysis discussed in the text. (**B**) Venn diagram quantifying the number of molecular ions detected from cultures of laboratory strains (PAO1 and PA14) compared to clinical isolates (nmFLRO1, mFLRO1, SH1B, SH2D, and SH3A). (**C**) UpSet plot quantifying the number of molecular ions detected from different combinations of cultures with colors highlighting strain-specific analyses discussed in the text.

Four hundred nine molecular ions were captured in the classical molecular network (Fig. 2A; Fig. S2). Under the experimental conditions, four members of the PHZ molecular family were detected, including PYO, 1-hydroxyphenazine (1-HP), phenazine-1-carboxamide (PCN), and phenazine-1-carboxylic acid (PCA). Forty-two AQs were detected, including HHQ, 2-heptyl-4-hydroxyquinoline *N*-oxide (HQNO), PQS, and their associated nonyl congeners (NHQ, NQNO, and C9-PQS). RLs produced by *P. aeruginosa* consist of two subfamilies, mono- and di-RLs, with one and two rhamnose units bound to 3‐(3‐hydroxyalkanoyloxy) alkanoic acids (HAAs), respectively. Both mono- and di-RLs were detected from various cultures, as was the siderophore PCH. The HSLs and PVDs were below the limit of detection under the extraction conditions used.

Three hundred thirty-three molecular ions (81%) were detected from the cultures of at least one laboratory strain and one clinical isolate (Fig. 2B), with 109 nodes (26%) measured from the cultures of all strains (Fig. 2C). Secondary metabolites detected from all strains included 1-HP, PCN, PCA, HHQ, HQNO, PQS, NHQ, NQNO, C9-PQS, and PCH. Notably absent from this set of secondary metabolites were the RLs and PYO. Seventeen nodes were detected in all strains except SH1B, including those representing RLs and PYO (Fig. 2C). Although most nodes in the network represented shared molecular ions between lab and clinical strain cultures, 74 nodes (18%) were detected solely from the cultures of clinical isolates (Fig. 2B), primarily from the cultures of mFLRO1 (20 nodes) and SH3A (14 nodes) (Fig. 2C). Only two nodes were measured solely from the cultures of the laboratory strains (Fig. 2B). These data illustrated that *P. aeruginosa* isolates have the capacity to produce the same suite of secondary metabolites as laboratory strains. However, some clinical isolates produce unique compounds and/or are unable to produce specific secondary metabolites under these culture conditions.

### Clinical isolate mFLRO1 produces acyl putrescines

**Fig 3:**
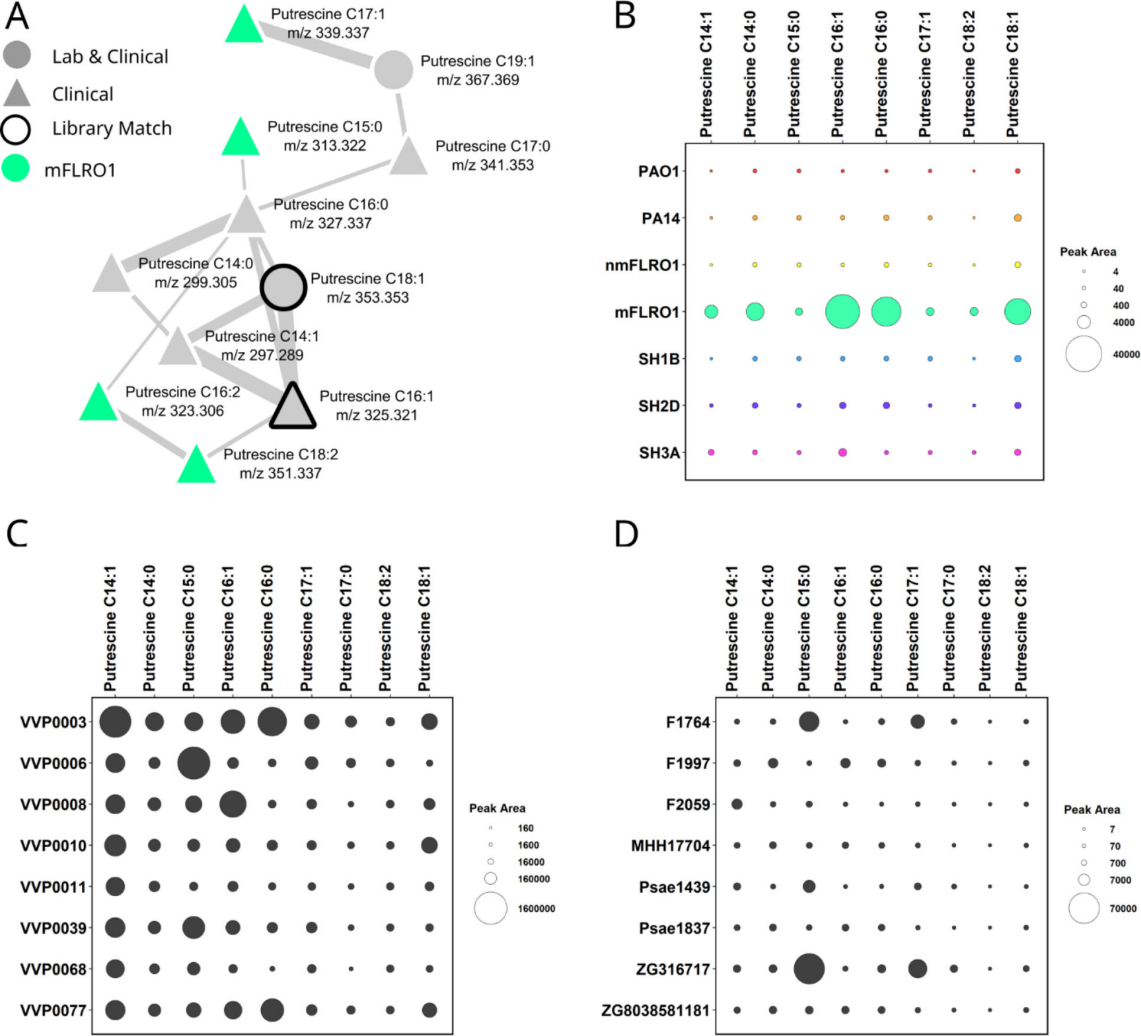
Acyl putrescine levels produced by *P. aeruginosa* isolates. (**A**) Acyl putrescine molecular family labeled with acyl chain length, unsaturation, and mass-to-charge ratio. Nodes represent molecular ions (precursor mass and MS/MS spectra). The node shape represents whether the molecular ion was detected from cultures of clinical isolates (triangle) or both laboratory strains and clinical isolates (circle). The node color indicates whether the molecular ion was detected in cultures from mFLRO1 (green) or combinations of strains (light gray). The widths of the lines connecting nodes (edges) represent the similarity of the MS/MS fragmentation of the connected nodes. Black node outlines indicate a spectral match between the data and MS/MS spectra within the GNPS spectral libraries. (**B**) Bubble plot representing the average quantitation (peak area) across biological replicates (*n* = 3) from each *P. aeruginosa* strain for the eight acyl putrescines above the limit of quantitation. Corresponding box plots for each metabolite are shown in Fig. S3. (**C**) Bubble plot representing the quantitation (peak area) of nine acyl putrescines from data set MSV000086107: publicly available LC-MS/MS data capturing the secondary metabolome of eight *P. aeruginosa* strains isolated from CF sputum cultured in Luria-Bertani (LB) broth under standard laboratory conditions (*n* = 1 biological replicate). (**D**) Bubble plot representing the quantitation (peak area) of nine acyl putrescines from eight *P. aeruginosa* strains from data set MSV000089869: publicly available LC-MS/MS data capturing the secondary metabolome of 35 *P. aeruginosa* isolates selected from the Helmholtz Centre for Infection Research Biobank, which were isolated from various sites of infection and cultured in LB broth under standard laboratory conditions (*n* = 1 biological replicate). Acyl putrescine levels from all 35 isolates are displayed in Fig. S6 in the supplemental material.

Twenty nodes in the classical molecular network represented molecular ions uniquely detected from the cultures of mFLRO1 (Fig. 2). Four of these nodes clustered within a molecular family of 11 members (Fig. 3A). Two nodes of the molecular family had spectral matches to the MS/MS spectra of acyl putrescine synthetic standards deposited within the GNPS libraries (Fig. S3) (50). Acyl putrescines consist of the polyamine putrescine conjugated to fatty acids of varying chain length and unsaturation. The two library matches putatively identified putrescine conjugated to hexadecenoic acid (putrescine C16:1) and octadecenoic acid (putrescine C18:1). These annotations were manually confirmed by comparing the experimental accurate mass and MS/MS measurements to the predicted and library values. The other nine members of the acyl putrescine molecular family were putatively annotated based on mass defect and MS/MS spectral similarity to the GNPS library matches (Table S2).

Of the 11 nodes within the acyl putrescine molecular family, 9 nodes were composed of spectra solely from the cultures of clinical isolates. To determine which of the isolates produced the highest quantity of acyl putrescines, their relative abundance was quantified and compared between strains (Fig. 3B; Fig. S4). Only eight of the acyl putrescines were measured above the limit of quantitation. Strain mFLRO1 produced the highest concentration of acyl putrescines, preferentially producing putrescine C16:1, putrescine C18:1, and putrescine conjugated to hexadecanoic acid (putrescine C16:0). To determine if the production of acyl putrescines was media dependent, the seven strains were cultured in Luria-Bertani (LB) broth (Fig. S5). Strain mFLRO1 produced the highest levels of acyl putrescines in LB broth, preferentially producing putrescine C16:1, putrescine C18:1, and putrescine C16:0. This result indicated that acyl putrescine production by *P. aeruginosa* is likely strain dependent but media independent.

To determine whether other *P. aeruginosa* isolates could produce acyl putrescines, two publicly available metabolomics data sets were downloaded from the MassIVE data repository and queried using MassQL (51, 52). Data sets MSV000086107 and MSV000089869 contain LC-MS/MS data that captured the secondary metabolome from LB media of eight *P. aeruginosa* clinical isolates collected at the University of California, San Diego Adult CF Clinic, and 35 isolates from various sites of infection selected from the Helmholtz Centre for Infection Research biobank, respectively (10, 40).

MassQL is a computational method that enables users to search publicly available metabolomics data for patterns captured in MS data that are intrinsically related to compound structure, such as isotopic ratio or compound class-specific fragments captured in the MS/MS spectra (52). The MS/MS spectra of data sets MSV000086107 and MSV000089869 were searched for the presence of two structural characteristics of acyl putrescines captured in their MS/MS spectra: a neutral loss corresponding to the mass of NH_2_ (−17.0264 Da) and a fragment ion of *m*/*z* 72.0802 corresponding to the molecular formula C_4_H_12_N^+^. This MassQL query resulted in the identification of acyl putrescines in both data sets. These results were manually verified by comparing experimental accurate mass and MS/MS measurements between the queried data set and the annotations of the acyl putrescines described above (Table S2).

The acyl putrescines were quantified from all samples within each data set using the built-in quantitation algorithm of GNPS Dashboard (53). GNPS Dashboard is a web-based platform for the visualization of open-source format LC-MS data. Chromatographic peak area for individual molecules is calculated in GNPS Dashboard using user-provided exact mass and retention time parameters. Importantly, due to differences in experimental conditions and data acquisition, intensity values could only be compared within each data set. Statistical comparison of acyl putrescine abundance between the isolates included in the data sets could not be performed as both data sets contained metabolomics data for single replicates.

Acyl putrescines were detected from all eight clinical isolates within data set MSV000086107, with VVP0003 and VVP0006 producing the highest relative quantities (Fig. 3C). Unlike mFLRO1, which predominantly produced putrescine C16:1, putrescine C18:1, and putrescine C16:0, the fatty acids incorporated into the acyl putrescines by the isolates of data set MSV000086107 varied in length and unsaturation. Isolate VVP0003 preferentially produced putrescine C14:0, putrescine 16:1, and putrescine 16:0; VVP0006 produced putrescine C15:0; and VVP0008 produced putrescine C16:1. Acyl putrescines were also detected in culture extracts of data set MSV000089869 (Fig. 3D; Fig. S6). Of the 35 isolates included in the study, strains F1764 (isolated from the respiratory tract) and ZG316717 (isolated from an ear infection) produced the highest levels of acyl putrescines, with both preferentially making putrescine C15:0 and putrescine C17:1. This result showed that different *P. aeruginosa* isolates produce acyl putrescines, with strain-dependent incorporation of different fatty acids.

Dysregulation of polyamine biosynthesis in *P. aeruginosa* clinical isolates is well documented (54–58). Putrescine is catabolized to succinate and ammonia through the putrescine utilization pathway and/or converted to spermidine by *P. aeruginosa*. Although these polyamines have been shown to enhance *P. aeruginosa* tolerance to antibiotic and oxidative damage by binding to lipopolysaccharide, high concentrations of spermidine are toxic. To control free spermidine levels, *P. aeruginosa* is known to *N-*acetylate spermidine (59, 60). Under our culture conditions, neither acetylated nor acylated spermidine was detected in mFLRO1 culture extracts. Production of acyl putrescines by *P. aeruginosa* isolates may be an alternative deactivation mechanism to control culture alkalinity due to putrescine degradation, as evidenced by the lower alkalinity of mFLRO1 cultures compared to the other isolates (Fig. 1C), and/or to reduce the toxicity associated with high free spermidine levels (61, 62).

### Clinical isolate SH3A is genetically related to PA-W1

**Fig 4:**
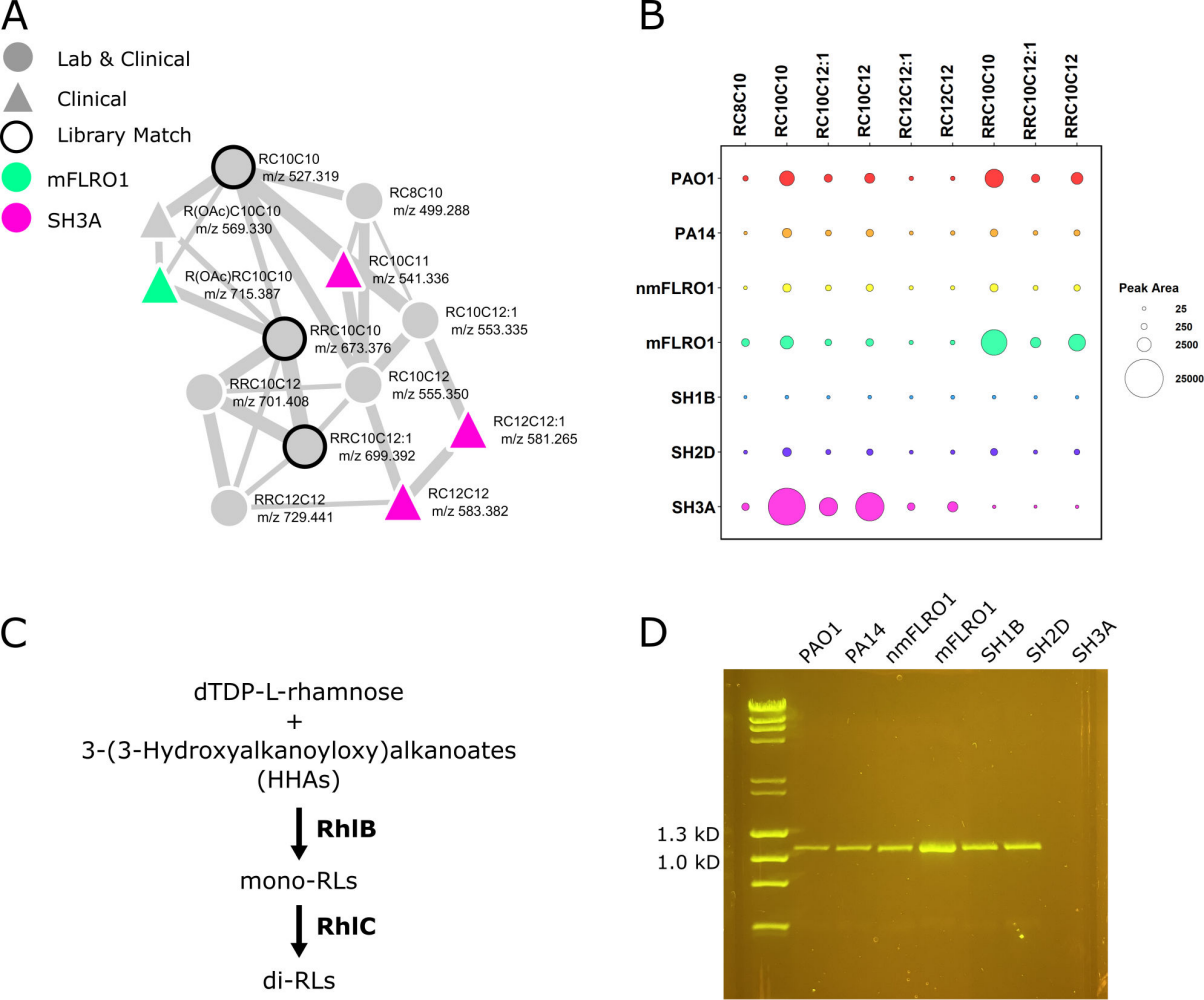
Rhamnolipid production by *P. aeruginosa* strains. (**A**) Rhamnolipid molecular family labeled with one or two rhamnose units (R or RR, respectively), acyl chain length, unsaturation, and mass-to-charge ratio. Nodes represent molecular ions (precursor mass and MS/MS spectra). The node shape represents whether the molecular ion was detected from cultures of clinical isolates (triangle) or both laboratory strains and clinical isolates (circle). The node color indicates whether the molecular ion was detected uniquely in cultures from SH3A (pink) or mFLRO1 (green), or combinations of strains (gray). The widths of the lines connecting nodes (edges) represent the similarity of the MS/MS fragmentation of the connected nodes. Black node outlines indicate a spectral match between the data and MS/MS spectra within the GNPS spectral libraries. (**B**) Bubble plot representing the average quantitation (peak area) of biological replicates (*n* = 3) from each *P. aeruginosa* strain for the nine rhamnolipids above the limit of quantitation. Corresponding box plots for each metabolite are shown in Fig. S6 in the supplemental materials. (**C**) Biosynthetic pathway of mono-RLs and di-RLs from precursors. (**D**) PCR analysis for the presence of *rhlC* in the genomes of the seven *P. aeruginosa* strains.

Fourteen nodes in the classical molecular network represented molecular ions uniquely detected from the cultures of SH3A (Fig. 2). Three of these nodes clustered within a molecular family of 13 members (Fig. 4A). Three nodes within the molecular family had spectral matches to the MS/MS spectra of characterized mono- and di-RLs RC10C10, RRC10C10, and RRC10C12:1 in the GNPS libraries (49). The other 10 members of the RL molecular family were annotated based on mass defect and MS/MS spectral similarity to the GNPS library matches (Table S2). This analysis led to the identification of seven mono-RLs and three di-RLs, including the discovery of one mono-RL and one di-RL with monoacetylated rhamnose units (Fig. S7).

The three nodes representing molecular ions in the RL molecular family detected from only SH3A cultures were annotated as three mono-RLs (RC10C11, RC12C12:1, and RC12C12). Further evaluation of the metadata information in category strain of the molecular family revealed that, although SH3A produced mono-RLs, di-RLs were not detected from SH3A cultures. To determine whether preferential production of mono-RLs was unique to SH3A, RL relative abundance was quantified and compared between strains (Fig. 4B; Fig. S8). Only nine of the RLs were measured above the limit of quantitation. Strain SH3A was the only strain to exclusively produce mono-RLs.

In *P. aeruginosa*, di-RLs are produced by the sequential functions of RhlA, RhlB, and RhlC (Fig. 4C). RhlA produces HAAs, which are subsequently glycosylated by the rhamnosyltransferase RhlB to form mono-RLs (63). RhlC condenses dTDP-L-rhamnose with mono-RLs to produce di-RLs. Although RhlA and RhlB are encoded in a bicistronic operon (*rhlAB*), RhlC is encoded separately on the *P. aeruginosa* genome (64). The inability of SH3A to produce di-RLs led to the hypothesis that SH3A did not have functional RhlC, resulting in the production of only mono-RLs. PCR analysis of the SH3A genome revealed that it did not encode for RhlC (Fig. 4D).

Recent phylogenetic analysis of thousands of genomes illustrated that *P. aeruginosa* strains segregate into five distinct phylogenetic clades, with the genomes of PAO1, PA14, PA7, and PA-W1 representing clades 1, 2, 3, and 5, respectively (65–67). Despite compelling evidence of the existence of clade 4, it has not yet been independently confirmed (65). Research has shown that the genomes of clades 1, 2, 3, and 5 can be distinguished from each other by the presence or absence of a subset of genes, including the absence of *rhlC* from clade 3 and 5 genomes (65–68). Therefore, we hypothesized that the absence of *rhlC* was an indicator the genome of SH3A belonged to clade 3 or 5 of the *P. aeruginosa* phylogenetic tree.

**Fig 5:**
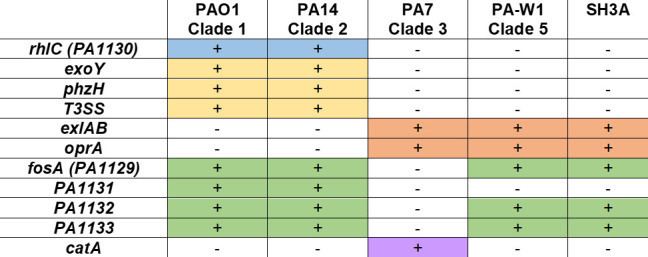
Genes distinguishing SH3A as a clade 5 isolate. Presence/absence analysis of differentiating genes between the genome of SH3A and representative genomes of clades 1 (PAO1), 2 (PA14), 3 (PA7), and 5 (PA-W1). Blue denotes rhlC; yellow denotes genes only in clade 1 and 2 genomes; orange denotes genes only in clade 3 and 5 genomes; green indicates genes that flank rhlC to distinguish between clade 3 and 5 genomes; purple indicates catA, only in clade 3 genomes.

To determine whether the genome of SH3A belonged to clade 3 or 5 of the *P. aeruginosa* phylogenetic tree, its genome was sequenced and the presence or absence of differentiating genes was evaluated between its genome and representative genomes of clades 1 (PAO1), 2 (PA14), 3 (PA7), and 5 (PA-W1) (Fig. 5). Comparative genomic analysis revealed that *rhlC* was indeed absent from the genome of SH3A (Fig. 5, blue). To verify that the loss of *rhlC* from the SH3A genome was not due to a singular event, the genome of SH3A was evaluated for the presence of *exoY*, *phzH*, genes of the type 3 secretion system (T3SS), *exlAB*, and *oprA*. The genes for ExoY, PhzH, and the T3SS are present in clade 1 and 2 genomes but absent from clade 3 and 5 genomes (Fig. 5, yellow). Conversely, the genes for ExlA, ExlB and OprA are present in clade 3 and 5 genomes but absent from clade 1 and 2 genomes (Fig. 5, orange). As *exlAB* and *oprA* were present in the SH3A genome but *exoY*, *phzH*, and *T3SS* were absent, this analysis confirmed that the SH3A genome belonged to clade 3 or 5 of the *P. aeruginosa* phylogenetic tree.

As the deletion of *rhlC* from the genomes of clades 3 and 5 was likely due to different evolutionary events, the genomes of these two clades can be distinguished from one another by the presence or absence of genes flanking *rhlC* (Fig. 5, green) (66). In clade 3 genomes, a five-gene deletion event led to the loss of *fosA* (*PA1129*), *rhlC* (*PA1130*), *PA1131*, *PA1132*, and *PA1133* compared to the PAO1 genome. In clade 5 genomes, a smaller deletion event occurred that led to the loss of *rhlC (PA1130*) and *PA1131* but left *fosA (PA1129*), *PA1132*, and *PA1133* intact. Evaluation of the SH3A genome for the presence or absence of the genes *fosA* (*PA1129*), *PA1131*, *PA1132*, and *PA1133* that flank *rhlC* (*PA1130*) indicated that the genes *fosA* (*PA1129*), *PA1132*, and *PA1133* were intact. Therefore, SH3A does not produce di-RLs because its genome belongs to clade 5 of the *P. aeruginosa* phylogenetic tree. This observation was confirmed by the absence of the clade 3-specific gene *catA* from the SH3A genome (Fig. 5, purple).

Strains belonging to phylogenetic clades 3–5 represent less than 1% of the sequenced genomes of *P. aeruginosa* and are genetically distinct from laboratory strains PAO1 and PA14, with some researchers advocating for these isolates to be considered a separate species (69). As these strains are poorly represented in publicly available genomic databases, screening the secondary metabolome of *P. aeruginosa* isolate libraries could be used to prioritize strains that putatively belong to the phylogenetic clades 3 and 5 for further analyses. As described above, the biosynthetic pathway for di-RLs consists of a bicistronic operon of *rhlAB* (*PA3478*/*PA3479*) and the accessory gene *rhlC* (*PA1130*) located separately on the genome (64). Analogously, the biosynthesis of the phenazine PCN is dependent upon the function of the core BGCs phz1 (*PA4210–PA4216*) and/or phz2 (*PA1899–PA1905*) and the accessory gene *phzH* (*PA0051*) located separately on the genome (70). Like *rhlC*, *phzH* is absent from clade 3 and clade 5 *P*. *aeruginosa* genomes. Since untargeted secondary metabolite profiling of *P. aeruginosa* captures both PHZs and RLs, secondary metabolite profiling could be used as a method to prioritize candidate strains that do not produce di-RLs and PCN but still produce mono-RLs and the other PHZs for genome sequencing. By prioritizing these strains for genome sequencing, more clade 3 and 5 genomes would be represented in the genome databases, enabling a more thorough phylogenetic comparison between isolates within these rare clades to understand their evolutionary diversion from clades 1 and 2.

### Clinical isolate SH1B genome has disrupted *rhlR*

**Fig 6:**
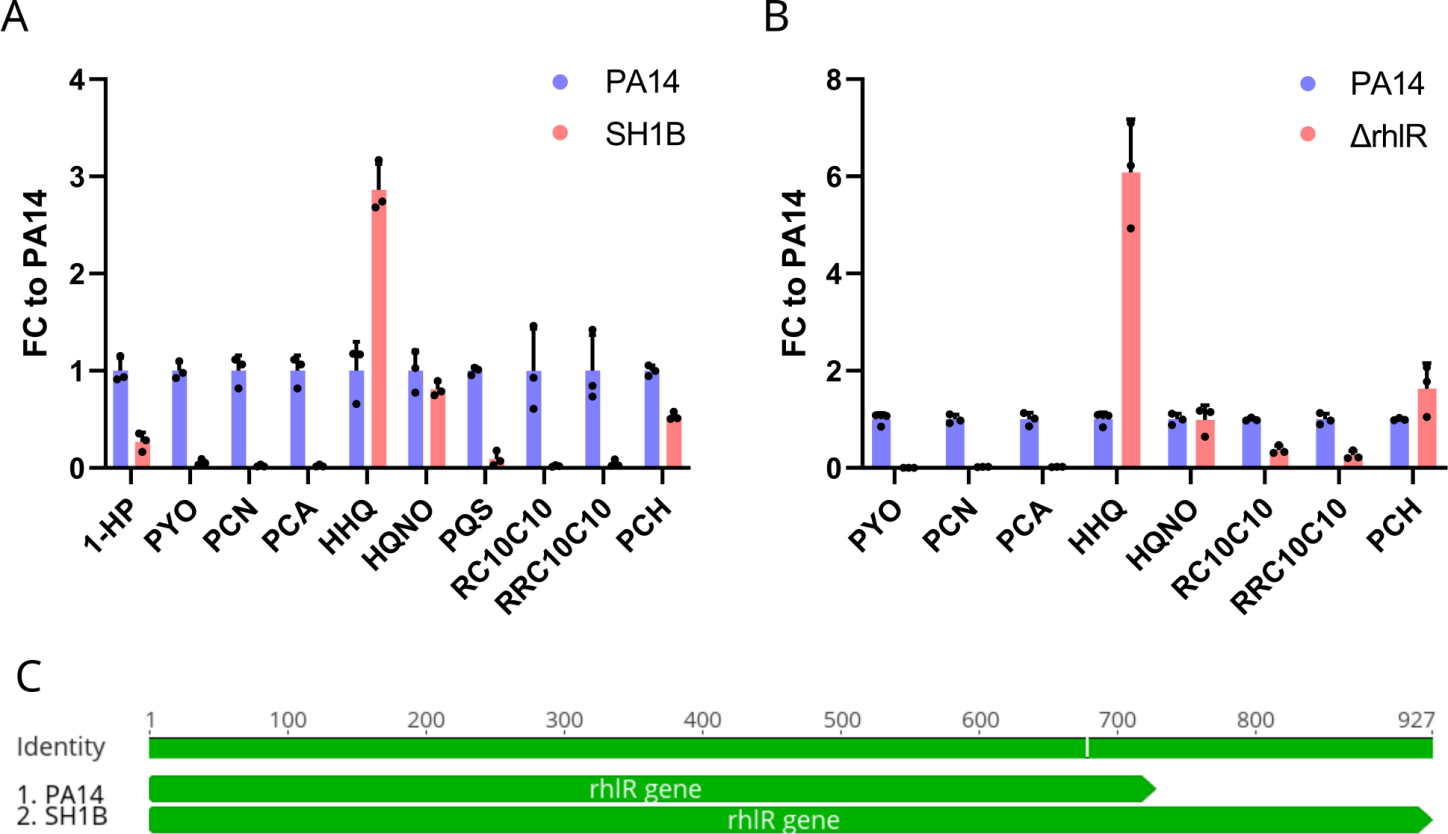
SH1B has disrupted *rhl* QS. (**A**) Ratio of secondary metabolite levels produced by SH1B compared to PA14 in SCFM2. Corresponding box plots for each metabolite are shown in Fig. S7. (**B**) Ratio of secondary metabolite levels produced by *ΔrhlR* compared to the PA14 parental strain in LB broth (MSV000083500). 1-HP and PQS were below the limit of detection and/or quantitation. Corresponding box plots for each metabolite are shown in Fig. S11. (**C**) The *rhlR* gene of SH1B is predicted to produce an elongated RhlR protein compared to PA14 due to a single-nucleotide insertion, which leads to a one-base frameshift and improper termination of translation. SH1B *rhlR* gene sequence alignment to only PA14 is shown for clarity. The corresponding gene sequence alignment to all strains in this study is shown in Fig. S12.

Seventeen nodes in the classical molecular network represented molecular ions that were detected from all cultures, except those of SH1B (Fig. 2). Three of these nodes clustered within the RL molecular family and were annotated as the mono-RLs RC10C10, RC10C12:1, and RC10C12. Further evaluation of the metadata information in category strain of the molecular family revealed that no RLs were detected from SH1B cultures. Although SH1B produced AQs and PCH, the PHZ PYO was not detected. To verify this observation, the secondary metabolites 1-HP, PYO, PCA, HHQ, HQNO, PQS, RC10C10, RRC10C10, and PCH were quantified from SH1B cultures and compared to levels from PA14 (Fig. 6A; Fig. S9). Indeed, SH1B produced markedly reduced levels of PHZs, RLs, and PQS compared to PA14 but continued to produce HHQ, HQNO, and PCH. To determine if this pattern of secondary metabolite production was media dependent, secondary metabolites were quantified from SH1B and PA14 cultures in LB broth (Fig. S10). Like SCFM2 cultures, SH1B in LB broth produced reduced PHZ and RL levels compared to PA14 but continued to produce HHQ. However, PCH was not detected from SH1B cultures in LB broth, likely due to the difference in iron concentration between the two media, with SCFM2 representing a lower iron environment.

The pattern of secondary metabolite production by SH1B was reminiscent of the results from the secondary metabolite profiling of an *rhlR* deletion mutant compared to its PA14 parental strain (71, 72). To confirm that the pattern of SH1B secondary metabolite production reflected the pattern of the *rhlR* deletion mutant, the publicly available metabolomics data set MSV000083500 was downloaded and processed. Data set MSV000083500 consists of LC-MS/MS data that captured the secondary metabolome of Δ*rhlR* and its parental strain PA14 cultured in LB broth in biological triplicate (72). Comparison of the secondary metabolite levels between Δ*rhlR* and wild-type (WT) PA14 indicated that although Δ*rhlR* produced reduced quantities of RLs and PHZs, it produced AQs and PCH at levels equivalent to or higher than WT (Fig. 6B; Fig. S11). The similarity of the secondary metabolite profile of SH1B to Δ*rhlR* led to the hypothesis that SH1B had disrupted *rhl* QS signaling.

To determine if the genome of *P. aeruginosa* isolate SH1B had a mutation in the *rhlR* gene, its genome was sequenced and the nucleotide sequence of the SH1B *rhlR* was compared to the sequences from the genomes of the other strains in this study. This analysis revealed that the SH1B *rhlR* gene sequence contained a one-base insertion frameshift mutation (678insC), which disrupts the stop codon and leads to improper termination of RhlR protein synthesis (Fig. 6C; Fig. S12). The SH1B *rhlR* is predicted to produce a protein consisting of 308 amino acids, which is 67 amino acids longer than the RhlR produced by most *P. aeruginosa* strains. Moreover, the sequence for the SH1B *rhlR* stop codon is within the nucleotide sequence of *rhlI*, fully abrogating *rhl* QS.

To evaluate the frequency of this mutation among *P. aeruginosa* isolates, the nucleotide and predicted amino acid sequences for SH1B *rhlR* were searched against the genome sequences of *P. aeruginosa* isolates deposited into NCBI and *Pseudomonas* Genome Database (73, 74). This analysis identified four other isolates with elongated *rhlR* gene sequences, including PA103 (isolated from sputum), BL22 (isolated from the human eye), PABL048 (isolated from blood), and PS1793 (isolated from sputum). The *rhlR* nucleotide sequences of SH1B, PA103, BL22, PABL048, and PS1793 are 100% identical (Fig. S13). This analysis indicated that the frameshift mutation identified in the SH1B *rhlR* gene sequence is rare (<0.0004%) in the sequenced genomes of *P. aeruginosa*. Taken together, the inability of SH1B to produce RLs and PYO but maintain production of AQs and PCH is due to a loss of function mutation in the *rhl* QS signaling pathway.

These results suggest that secondary metabolite profiling of *P. aeruginosa* isolates could be used to screen isolate libraries for mutants with defective QS pathways. Mutations within the QS signaling pathways of *P. aeruginosa* frequently occur, with mutations in *lasR* more common than mutations in *rhlR* (71). The *las* QS system controls a regulon of virulence-associated genes, including the biosynthesis of all secondary metabolite molecular families (3). Therefore, *P. aeruginosa* isolates with true loss of *lasR* function would be expected to be unable to produce any secondary metabolites. However, some isolates with *lasR* mutations are still able to produce QS-regulated virulence factors (75). In evolution experiments of laboratory strain *P. aeruginosa* PAO1, mutations within the gene of virulence-associated transcriptional regulator MexT recovered RL and PYO biosynthesis in isolates with a loss of function *lasR* mutation, providing an explanation for the origin of LasR-independent *rhl* QS pathway activation (31). As a result, *P. aeruginosa* isolates with predicted LasR and/or RhlR loss of function mutations could be screened for the absence of the production of specific secondary metabolite classes, enabling the separation of true loss of QS function isolates from those that have escape mutations in *mexT* or other regulatory genes and providing additional insight into the association of genotypes with virulence to complement existing targeted analysis of HSLs and AQs (32).

### Conclusion

In this work, we used untargeted LC-MS/MS to profile the secondary metabolome of seven *P. aeruginosa* strains, including five clinical isolates (nmFLRO1, mFLRO1, SH1B, SH2D, and SH3A) from the sputum of people with CF and two laboratory strains (PAO1 and PA14). By analyzing the data with CMN, we quantified the commonalities and differences in secondary metabolite production between *P. aeruginosa* isolates and laboratory strains. This analysis revealed that *P. aeruginosa* isolates and laboratory strains have the genetic capacity to produce a common secondary metabolome, but the ability to produce the different secondary metabolite molecular families is dictated primarily by the genome sequence of the strain under study. We identified that some *P. aeruginosa* isolates, including mFLRO1, can produce a previously undescribed class of acyl putrescines and posit the production of this molecular family is a mechanism to deactivate putrescine degradation to the toxic product spermidine. Genomic sequencing of SH3A illustrated that its lack of di-RL production was indicative of its genome belonging to clade 5 of the *P. aeruginosa* phylogenetic tree, which represents <1% of publicly available sequenced genomes. Using secondary metabolite profile similarity to Δ*rhlR*, we discovered that the genome of SH1B has a loss of function mutation in RhlR that disrupts *rhl* QS signaling, resulting in the loss of RL and PYO biosynthesis, a mutation that was only found in four of thousands of publicly available genome sequences. These results indicate that secondary metabolite profiling of *P. aeruginosa* isolates can provide unique insight into their genomic variability. Considering that this study profiled the secondary metabolome of five *P. aeruginosa* clinical isolates and captured three rare genetic traits representing <1%–5% of the population with publicly available genomes or metabolomes, it raises the question of how truly infrequent these traits are in the population and whether the isolates captured in the current databases accurately represent the genomic diversity of *P. aeruginosa*. Integration of secondary metabolite profiling in future investigations of *P. aeruginosa* population diversity would aid in the prioritization of rare genotypes for sequencing, discern the effect of mutations on QS pathways, and identify biomarkers of virulence.

## MATERIALS AND METHODS

### Strains

The *P. aeruginosa* strains used in this study are summarized in Table S1.

### Culture of *P. aeruginosa* in SCFM2

SCFM2 was prepared as previously described with the following alteration: commercial bovine submaxillary mucin type I-S (BSM, Millipore Sigma) was dialyzed prior to addition to the medium (42). Briefly, BSM was suspended in 1× 3-morpholinopropane-1-sulfonic acid (MOPS) buffer (pH 7.0), dialyzed against 1× MOPS using a 10-kDa molecular weight cutoff cassette (Thermo Fisher Scientific), and sterilized using a liquid autoclave cycle (15-min sterilization time) prior to addition to the medium. SCFM2 was stored in the dark at 4°C, checked for sterility prior to use, and used within 1 month of preparation. *P. aeruginosa* was inoculated from streak plates into 5 mL LB broth and incubated overnight at 37°C, shaking at 220 RPM. SCFM2 (0.990 mL) was inoculated with 10 µL of *P. aeruginosa* LB culture (OD600 of 0.05, ~1 × 10^6^ CFU/mL) in a polystyrene 48-well plate. The plate was covered with its lid and incubated statically, under ambient oxygen conditions, at 37°C for 48 h.

### Sample processing

Gross phenotypes of SCFM2 cultures were photographed with a top-down view of each well using a Stemi 508 stereo microscope with an Axiocam 105 color camera (Zeiss). Samples were mechanically disrupted by pipetting. Disrupted samples were aliquoted for growth measurements (CFU per milliliter), culture pH, and metabolomics analysis. All biological replicates (*n* = 3) were serially diluted, spotted onto LB agar, incubated at 37°C overnight, and counted to determine colony-forming units by volume (CFU per milliliter).

### Sample preparation for metabolomics analysis

Each sample was chemically disrupted with an equal volume of 1:1 solution of ethyl acetate (EtOAc, VWR HiPerSolv Chromanorm) and methanol (MeOH, Fisher Scientific Optima LC/MS grade). The samples were dried and stored at −20°C until use. After thawing, samples were resuspended in 100% MeOH, diluted fivefold in 100% MeOH containing 1 µM glycocholic acid (Calbiochem, 100.1% pure), and centrifuged for 10 min at 4,000 RPM (Thermo Sorvall ST 40R) to remove non-soluble particulates prior to injection.

### LC-MS/MS data acquisition

Mass spectrometry data acquisition was performed using a Bruker Daltonics Maxis II HD qTOF mass spectrometer equipped with a standard electrospray ionization (ESI) source as previously described (7). The mass spectrometer was tuned by infusion of Tuning Mix ESI-TOF (Agilent Technologies) at a 3-µL/min flow rate. For accurate mass measurements, a wick saturated with Hexakis (1H,1H,2H-difluoroethoxy) phosphazene ions (Apollo Scientific, *m*/*z* 622.1978) located within the source was used as a lock mass internal calibrant. Samples were introduced by an Agilent 1290 UPLC using a 10-µL injection volume. Extracts were separated using a Phenomenex Kinetex 2.6-µm C18 column (2.1 × 50 mm) using a 9-min linear water-acetonitrile (ACN) gradient (from 98:2 to 2:98 water:ACN) containing 0.1% formic acid at a flow rate of 0.5 mL/min. The mass spectrometer was operated in data-dependent positive ion mode, automatically switching between full-scan MS and MS/MS acquisitions. Full-scan MS spectra (*m*/*z* 50–1500) were acquired in the time of flightTOF, and the top five most intense ions in a particular scan were fragmented via collision-induced dissociation using the stepping function in the collision cell. LC-MS/MS data for *P. aeruginosa* (PA) mix, a mixture of available commercial standards of *P. aeruginosa* secondary metabolites, were acquired under identical conditions. Bruker Daltonics CompassXport was used to apply lock mass calibration and convert the LC-MS/MS data from .d format to .mzXML format.

### CMN

The Molecular Networking workflow (version release 26) was applied to the .mzXML files using the GNPS analysis platform (45). Briefly, the data were filtered by removing all MS/MS fragment ions ± 17 Da of the precursor *m*/*z*. MS/MS spectra were window filtered by choosing only the top six fragment ions in each ±50-Da window throughout the spectrum. The precursor ion mass tolerance was set to 0.05 Da, and the MS/MS fragment ion tolerance was set to 0.1 Da. Each node was set to consist of at least five matching spectra with a minimum of five matched fragment ions between nodes to generate an edge. A network was then created where edges between nodes were filtered to have a cosine score above 0.8. Furthermore, edges between two nodes were kept in the network only if each of the nodes appeared in each other’s respective top 10 most similar nodes. Finally, the maximum size of a molecular family was set to 80, and the lowest scoring edges were removed until each molecular family was below this threshold. The spectra in the network were then searched against the GNPS spectral libraries. Library spectra were filtered in the same manner as the input data. All matches kept between the network spectra and library spectra were required to have a cosine score above 0.7 and at least five matched peaks.

### Venn diagram and UpSet plot generation

The network table generated via CMN was downloaded from Cytoscape (76). A binary presence/absence table was created from the network table to determine whether each node was detected in the cultures of each strain according to the GNPSGROUP designations. The UpSet plot was generated in R using the UpSetR package (77).

### Secondary metabolite quantitation from CF isolates

MZmine (version 2.53) was used to perform feature finding on the .mzXML files (78). The output files included a feature table containing area under the curve (AUC) integration values for each feature (*m*/*z*-RT pair) and an .mgf file containing MS/MS fragment ions for each feature. Features were normalized by row sum, and the mean aggregation was applied to peak abundances for each group. Features were filtered for rhamnolipids, acyl putrescines, and *P. aeruginosa* secondary metabolites using exact mass and retention time.

### Secondary metabolite analysis of PA14 and Δ*rhlR*

The publicly available *P. aeruginosa* secondary metabolite profiling data set MSV000083500 was downloaded from MassIVE (51, 72). MZmine (version 2.53) was used to perform feature finding on the .mzML files (78). The output files included a feature table containing AUC integration values for each feature (*m*/*z*-RT pair) and an .mgf file containing MS/MS fragment ions for each feature. Features were filtered for known *P. aeruginosa* metabolites.

### Metabolite annotation

The classical molecular network (https://gnps.ucsd.edu/ProteoSAFe/status.jsp?task=de0b3f2b01174831b5a2be8546812ca1) was visualized using Cytoscape (version 3.7.1) (76). Extracted ion chromatograms of *m*/*z* values of interest were visually inspected using the GNPS Dashboard (https://dashboard.gnps2.org/), and MS/MS spectra were visualized using the Metabolomics Spectrum Resolver (https://metabolomics-usi.gnps2.org/) (53, 79). All annotated nodes corresponding to *P. aeruginosa* specialized metabolites identified from CMN are listed in Table S2. Annotations of metabolites corresponding to commercial standards (level 1 annotation) were confirmed by comparing the experimental data (exact mass, MS/MS, and retention time) with data acquired for the compound in the PA mix using Bruker Daltonics DataAnalysis (version 4.1, Build 362.7) (80). Putative annotation of metabolites of interest corresponding to matches to the GNPS libraries (level 2 annotation) was validated by comparing the experimental data (exact mass, MS/MS) to reported data and putative structures, respectively (45, 48, 49, 80).

### MassQL query of public metabolomics data sets for acyl putrescines

Two published, publicly available *P. aeruginosa* secondary metabolite profiling data sets, MSV000086107 and MSV000089869, were downloaded from MassIVE to a GNPS workspace (10, 40, 45, 51). These data sets, along with our own data set as a positive control, were queried using MassQL v31.4 with the query:

QUERY scaninfo(MS2DATA) WHERE MS2PROD = 72.0802:TOLERANCEMZ = 0.005 AND MS2NL = 17.0264:TOLERANCEMZ = 0.005 (52). The results of the MassQL (version 31.4) query (https://gnps.ucsd.edu/ProteoSAFe/status.jsp?task=23c630b0fc484582a5e0187c6ce5ef99) were subsequently networked using the Classical Molecular Networking workflow (version 30) within GNPS (https://gnps.ucsd.edu/ProteoSAFe/status.jsp?task=f4284d9fa3f14ef6bceb6cbad0580033). The results were filtered using library matches to synthetic acyl putrescine standards in the GNPS libraries and precursor *m*/*z* values of the manually annotated acyl putrescines. To identify which isolates produced acyl putrescines, the chromatographic peak for each of the identified putrescines was identified in individual files using GNPS Dashboard, and the putative annotations were verified by manual inspection of the associated MS/MS spectra (53, 79). Peak areas of the acyl putrescines were quantified across each data set using the built-in quantitation algorithm of GNPS Dashboard with the parameters of exact mass of the eight-acyl putrescine with a 0.05-Da window and a retention time window of 30 seconds spanning peak maxima. The quantified values were visualized as bubble plots using R.

### PCR of 16S rRNA and *rhlC* genes

Genomic DNA was extracted from *P. aeruginosa* strains using the QIAamp DNA Mini Kit (Qiagen) according to manufacturer’s instructions. PCR-based detection of the 16S rRNA (positive control) and *rhlC* genes was completed using the Phusion High-Fidelity PCR kit (Thermo Fisher Scientific) following the manufacturer’s instructions with published primer sequences (64, 81). Gel electrophoresis was performed on all samples using a 1.0% agarose gel in 1× Tris-acetate-EDTA (TAE) buffer and visualized using SYBR Safe DNA Gel Stain (Thermo Fisher Scientific).

### Genome sequencing

*P. aeruginosa* CF isolates were inoculated from streak plates into 5 mL LB broth and incubated overnight at 37°C, shaking at 220 RPM. Genomic DNA was extracted using the QIAamp DNA Mini Kit (Qiagen) according to manufacturers’ instructions, and concentration and quality were confirmed by NanoDrop spectroscopy. Genomic sequencing using short-read Illumina and long-read Oxford Nanopore Technologies (ONT), *de novo* assembly, and annotation was performed by SeqCenter (Pittsburgh, PA). Briefly, Porechop (version 0.2.4) was used to trim residual adapter sequence from the ONT reads using default parameters that may have been missed during base calling and demultiplexing (82). *De novo* genome assemblies were generated from the ONT read data with Flye (version 2.9.2) under the nano-hq (ONT high-quality reads) model (--asm-coverage 50 --genome-size 6000000) (83). Additional Flye options initiate the assembly by first using reads longer than an estimated N50 based on a genome size of 6 Mbp. Subsequent polishing used the Illumina read data with Pilon (version 1.24) under default parameters (84). To reduce erroneous assembly artifacts caused by low-quality nanopore reads, long-read contigs with an average short-read coverage of 15× or less were removed from the assembly. Assembled contigs were evaluated for circularization via Circulator (version 1.5.5) using the ONT long reads (85). Assembly annotation was then performed with Bakta (version 1.8.1) using the Bakta (version 5) database (86). Assembly statistics were recorded with QUAST (version 5.2.0) (87).

### Genomic comparisons

Assembled and annotated genomes of the CF isolates included in this study were visualized in Geneious (version 2023.2.1) (88). To determine if the genome of SH3A belonged to clade 3 or 5 of the *P. aeruginosa* phylogenetic tree, genomes of PAO1, PA14, PA7, and PA-W1 were downloaded from NCBI and used as reference genomes for clades 1, 2, 3, and 5, respectively (73). Full genomic alignments were conducted using the Mauve plug-in with default parameters (89). Annotations of aligned genomes were searched for *exoY*, *phzH*, *rhlC*, *exlA*, *exlB*, *oprA*, *catA*, *fosA*, *PA1131*, *PA1132*, and *PA1133* and the gene cluster for T3SS. To determine if *rhlR* of SH1B contained a mutation that would disrupt *rhl* QS signaling, the nucleotide and predicted protein sequences were extracted from the genomes of PAO1, PA14, nmFLRO1, mFLRO1, SH1B, SH2D, and SH3A and aligned using Clustal Omega (version 1.2.2) with default parameters (90). The nucleotide and predicted protein sequences of SH1B *rhlR* were subsequently used for a Blast search in NCBI and the *Pseudomonas* Genome Database to identify other isolates with the same mutation (73, 74). The nucleotide sequences of *rhlR* from isolates BL22, PA103, PABL048, and PS1793 were downloaded. The nucleotide and predicted protein sequences of *rhlR* from these four isolates were aligned to *rhlR* from SH1B and PAO1 using Clustal Omega (version 1.2.2) with default parameters (90).

### Statistical analysis

Statistical comparison of metabolite abundance was conducted in GraphPad Prism (version 9.3.1). Unpaired two-tailed *t*-tests of differential secondary metabolite abundance between PA14 and SH1B and PA14 and Δ*rhlR* are summarized in the supplemental figure legends. For all analyses, *P* values of <0.05 were considered statistically significant.

## Data Availability

Mass spectrometry data files are available via MassIVE as MSV000087157. Genome sequences for nmFLRO1, mFLRO1, SH1B, SH2D, and SH3A are available in National Center for Biotechnology Information under BioProject PRJNA1077317.

## SUPPLEMENTAL MATERIAL

Supplemental Material msystems.00339-24-s0001.docx Supplemental tables and figures.

ASM does not own the copyrights to Supplemental Material that may be linked to, or accessed through, an article. The authors have granted ASM a non-exclusive, world-wide license to publish the Supplemental Material files. Please contact the corresponding author directly for reuse.
