# Supplementary material for: Secondary metabolite profiling of Pseudomonas aeruginosa isolates reveals rare genomic traits
Source: mSystems. 2024 Apr 15;9(5):e00339-24. doi: 10.1128/msystems.00339-24 (PMC11097636; doi:10.1128/msystems.00339-24)
Supplement: Supplemental Material — Supplemental tables and figures. [file msystems.00339-24-s0001.docx]

Supplementary Material for

“Classical molecular networking of the secondary metabolome of *Pseudomonas aeruginosa* isolates reveals rare genomic traits”

Rachel L. Neve^1,*^, Emily Giedraitis^2,*^, Madeline Akbari^1^, Shirli Cohen^1^, Vanessa V. Phelan^2#^

**AUTHOR AFFILIATIONS:**

^1^ Department of Immunology and Microbiology, School of Medicine, University of Colorado - Anschutz Medical Campus, Aurora, CO, 80045, USA

^2^ Department of Pharmaceutical Sciences, Skaggs School of Pharmacy and Pharmaceutical Sciences, University of Colorado - Anschutz Medical Campus, Aurora, CO, 80045, USA

*Authors contributed equally to the study

**CORRESPONDING AUTHOR:**

^#^ Address correspondence to Vanessa V. Phelan, vanessa.phelan@cuanschutz.edu

Table of Contents

[Table S1. *P. aeruginosa* strains used in this study. 3](#_Toc158710780)

[Figure S1. Replicate cultures of isolates in SCFM2 3](#_Toc158710781)

[Figure S2. Full classical molecular network of isolates in SCFM2 4](#_Toc158710782)

[Figure S3. MS/MS comparison of acyl putrescines from mFLRO1 and GNPS library 5](#_Toc158710783)

[Figure S4. Comparison of acyl putrescine abundance between isolates in SCFM2 6](#_Toc158710784)

[Figure S5. Comparison of putrescine C16:0 abundance between isolates in LB 6](#_Toc158710785)

[Figure S6. Bubble plot of acyl putrescine abundance of 35 strains from MSV000089869 7](#_Toc158710786)

[Figure S7. MS/MS annotation of acetylated rhamnolipids from isolates in SCFM2 8](#_Toc158710787)

[Figure S8. Comparison of rhamnolipid abundance between isolates in SCFM2 9](#_Toc158710788)

[Figure S9. Secondary metabolite abundance of PA14 v SH1B in SCFM2 10](#_Toc158710789)

[Figure S10. Ratio of secondary mteabolite levels producted by PA14 v SH1B in LB 10](#_Toc158710790)

[Figure S11. Secondary metabolite abundance of PA14 v ΔrhlR from MSV000083500 11](#_Toc158710791)

[Figure S12. Gene sequence alignment of rhlR of strains in this study 12](#_Toc158710792)

[Figure S13. Gene sequence alignment of rhlR of strains identified in database and PAO1 13](#_Toc158710793)

[Table S2. Secondary metabolite annotations 14](#_Toc158710794)

# Table S1. *P. aeruginosa* strains used in this study.

| **Strain** | **Origin** | **Other names** | **Reference/Source** |
| --- | --- | --- | --- |
| PAO1 | Lab strain | MPAO1 | University of Washington (1) |
| PA14 | Lab strain |  | Suzanne Noble, UCSF (2) |
| nmFLRO1 | CF isolate | PAnmFLR01 | Forest Rohwer, SDSU (3) |
| mFLRO1 | CF isolate | PAmFLR01 | Forest Rohwer, SDSU (3) |
| SH1B | CF isolate | 10/92 | Leo Eberl, UZH (4) |
| SH2D | CF isolate | 8/93 | Leo Eberl, UZH (4) |
| SH3A | CF isolate | 9/95 | Leo Eberl, UZH (4) |


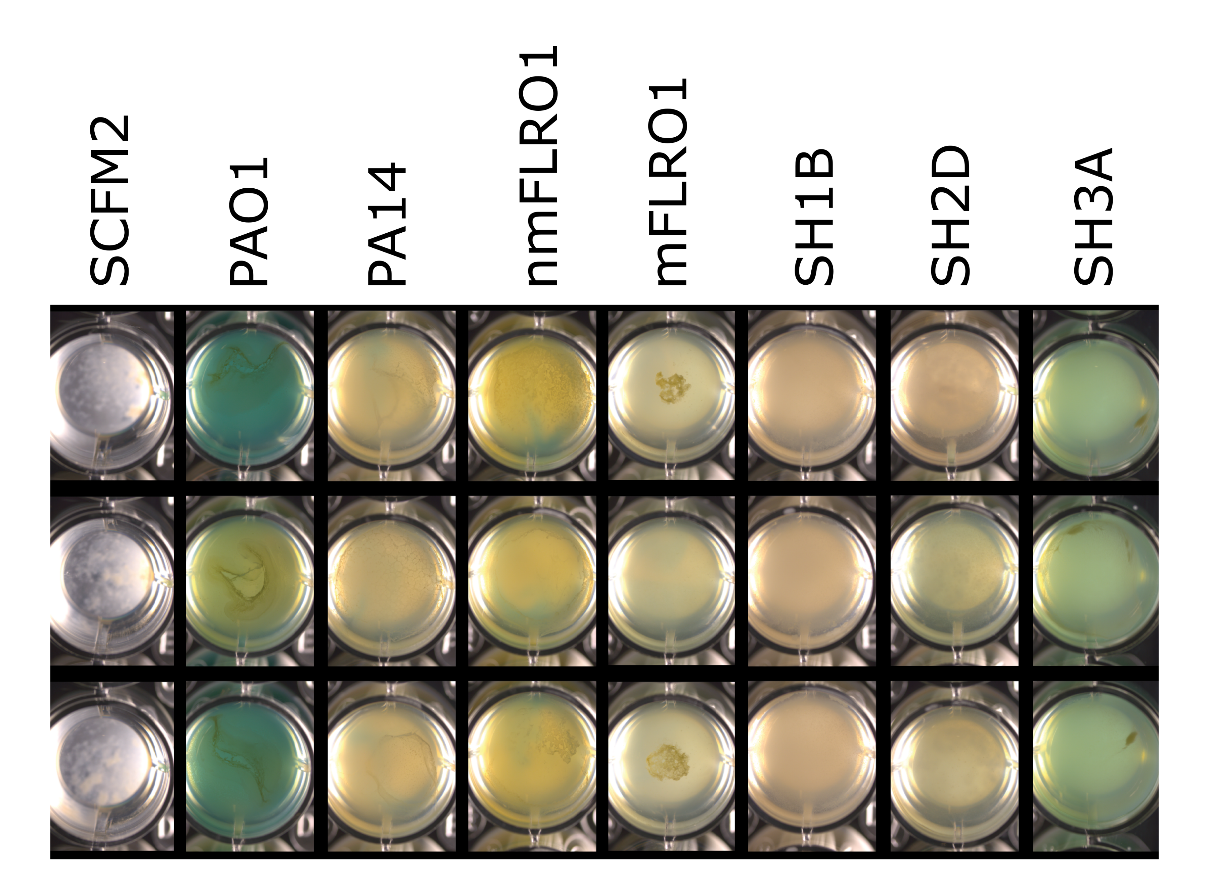


Figure S1. Growth of P. aeruginosa strains in SCFM2. Photographs of replicate control media wells and growth in SCFM2.

*
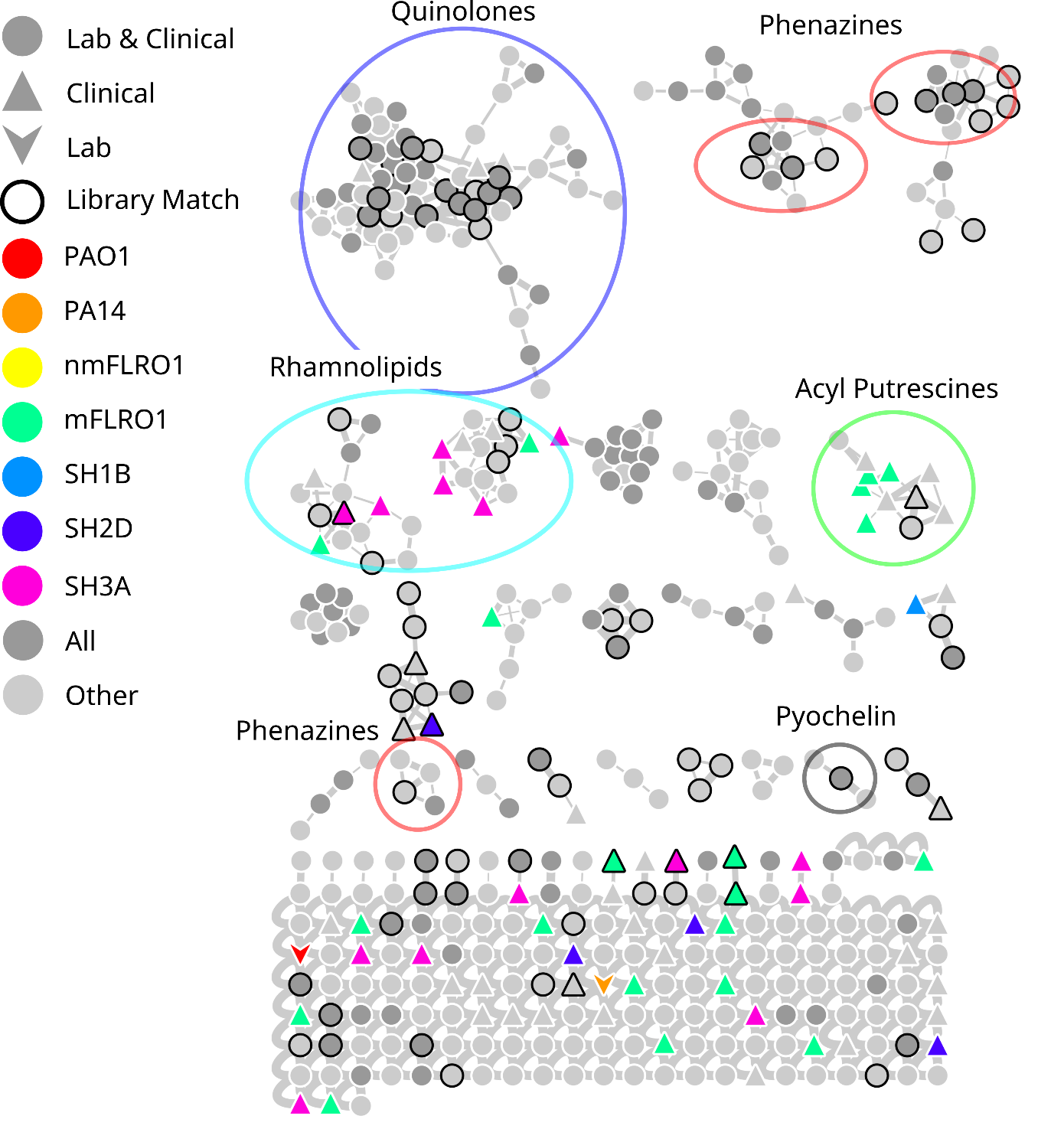
*

Figure S2. Classical molecular network of seven *P. aeruginosa* strains cultured in SCFM2. Nodes represent molecular ions (precursor mass and MS/MS spectra). The widths of the lines connecting nodes (edges) represent the similarity of the MS/MS fragmentation of the connected nodes. Nodes with black outlines indicate a spectral match between the data and MS/MS spectra within the GNPS spectral libraries. Node shape represents whether the molecular ion was detected from cultures of laboratory strains (down arrow), clinical isolates (triangle), or both (circle). Node color indicates whether the molecular ion was detected in cultures from all strains (dark gray) or individual strains (various colors), or combinations of strains (light gray). Nodes corresponding to the five molecular families discussed in the main text are highlighted by ovals.


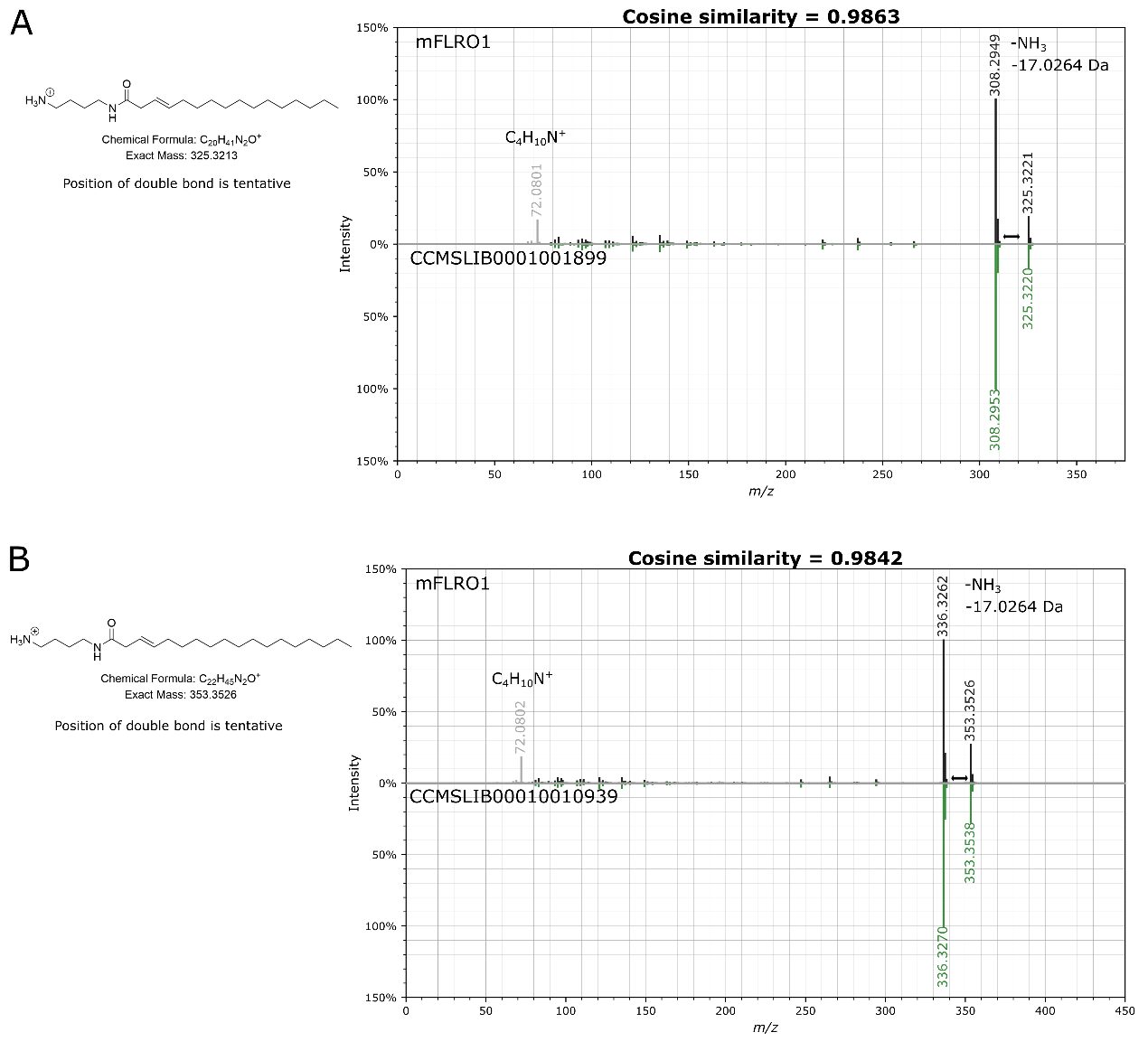


Figure S3. Comparison of MS/MS spectra between data collected from mFLRO1 cultures and GNPS spectral libraries for Putrescine C16:1 (A) and Putrescine C18:1 (B).


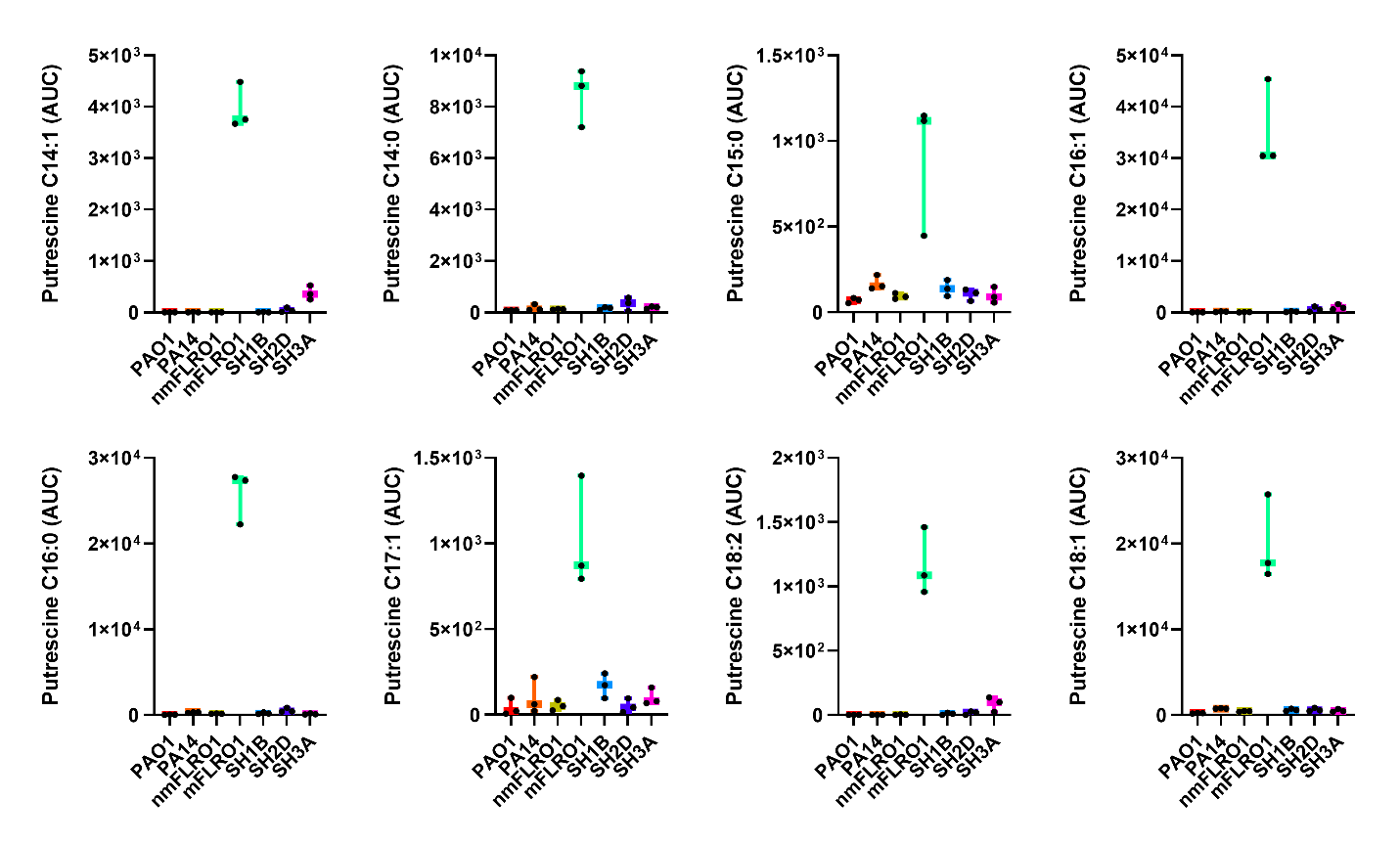


Figure S4. Differential levels of acyl putrescine congeners produced by seven P. aeruginosa strains in SCFM2. Box plots represent the 25 to 75th percentiles, with a line at the median. Error bars indicate the minimum to maximum. Individual sample values shown (n = 3 biological replicates per strain).


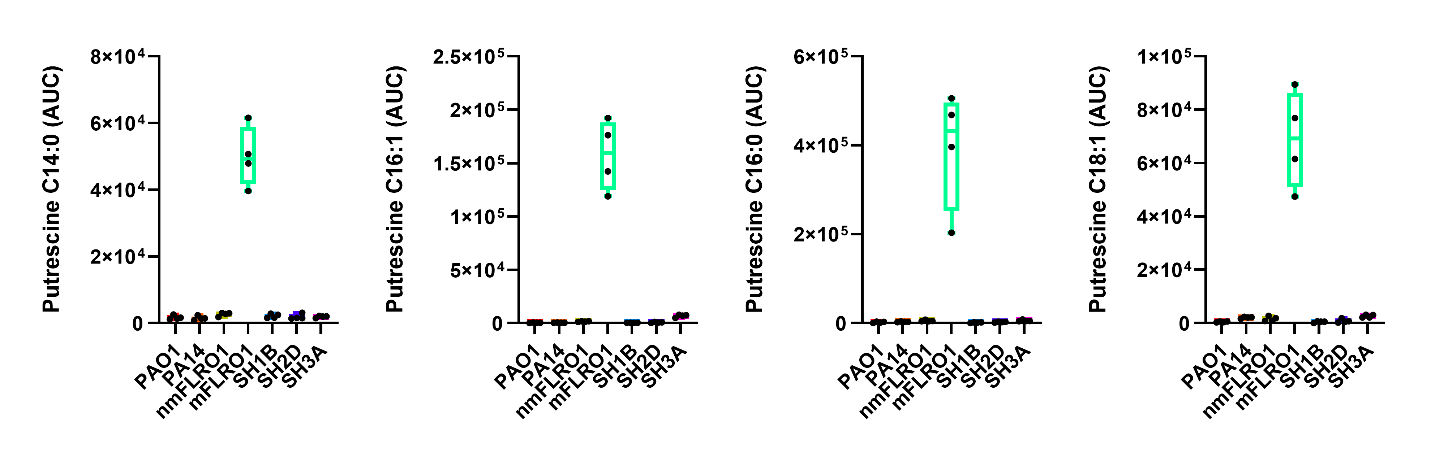


Figure S5. Differential levels of acyl putrescines produced by seven P. aeruginosa strains in LB. Box plots represent the 25 to 75th percentiles, with a line at the median. Error bars indicate the minimum to maximum. Individual sample values shown (n = 4 biological replicates per strain).


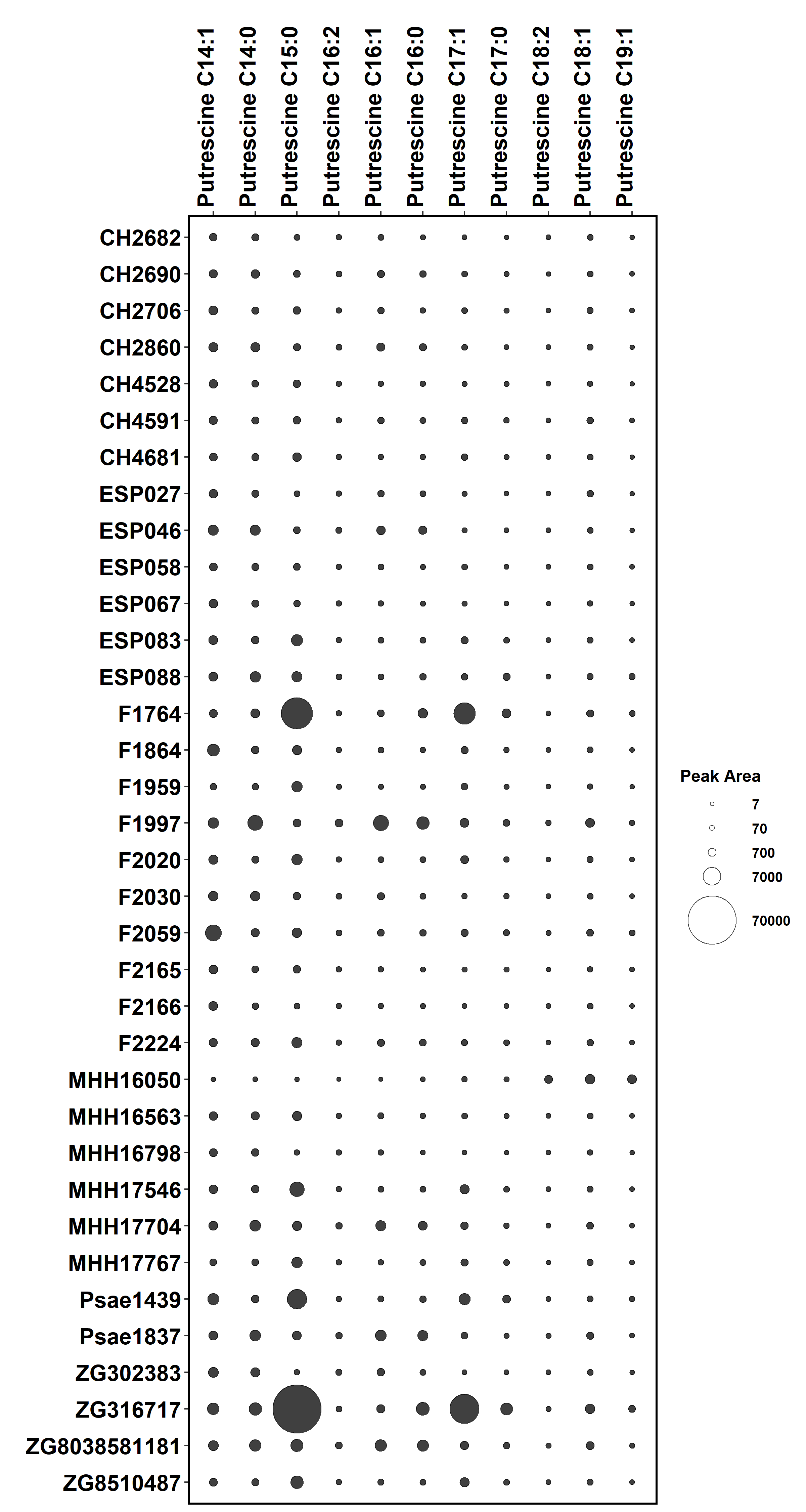


Figure S6. Bubble plot representing the quantitation (peak area) of eleven acyl putrescines from Dataset 2 (MSV000089869): publicly available LC-MS/MS data capturing the secondary metabolome of 35 P. aeruginosa isolates selected from the Helmholtz Centre for Infection Research biobank which were isolated from various sites of infection and cultured in LB under standard laboratory conditions (n = 1 biological replicate).


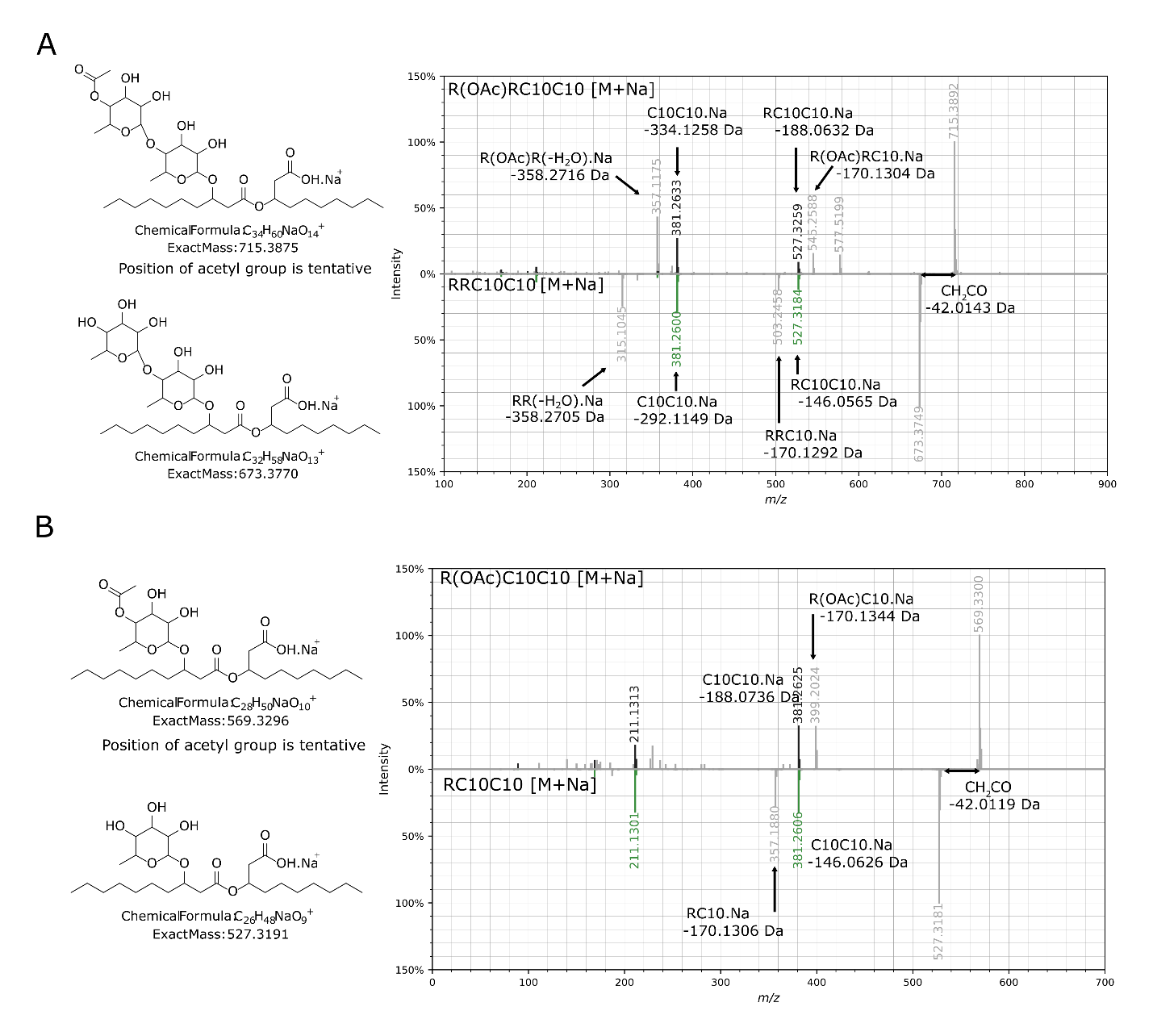


Figure S7. Annotation of the MS/MS spectra for the acetylated rhamnolipids (A) R(OAc)RC10C10 and (B) R(OAc)C10C10 compared to structurally characterized rhamnolipids RRC10C10 and RC10C10. Localization of acetyl group is putative.


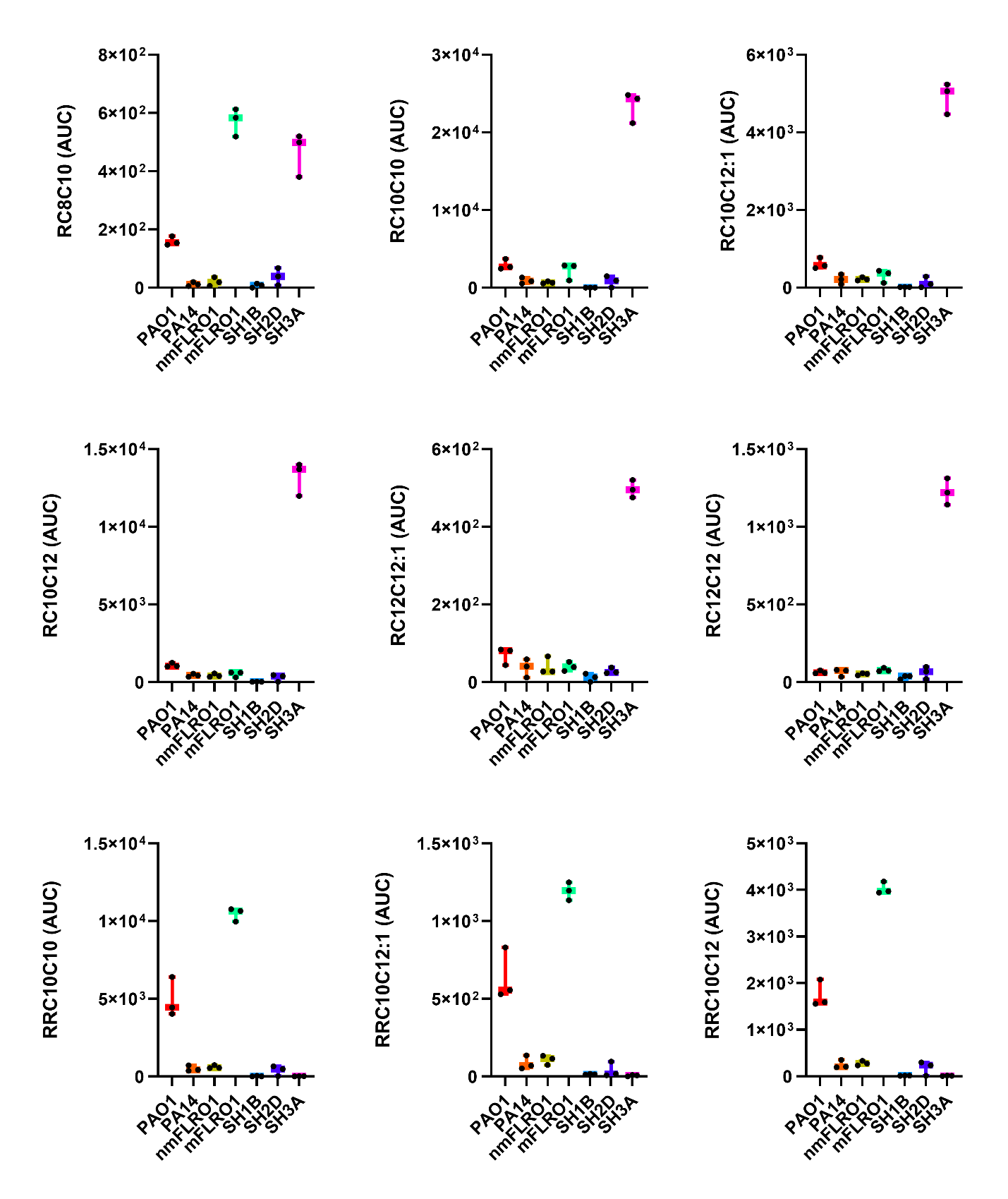


Figure S8. Differential levels of rhamnolipids produced by seven P. aeruginosa strains in SCFM2. Box plots represent the 25 to 75th percentiles, with a line at the median. Error bars indicate the minimum to maximum. Individual sample values shown (n = 3 biological replicates per strain).


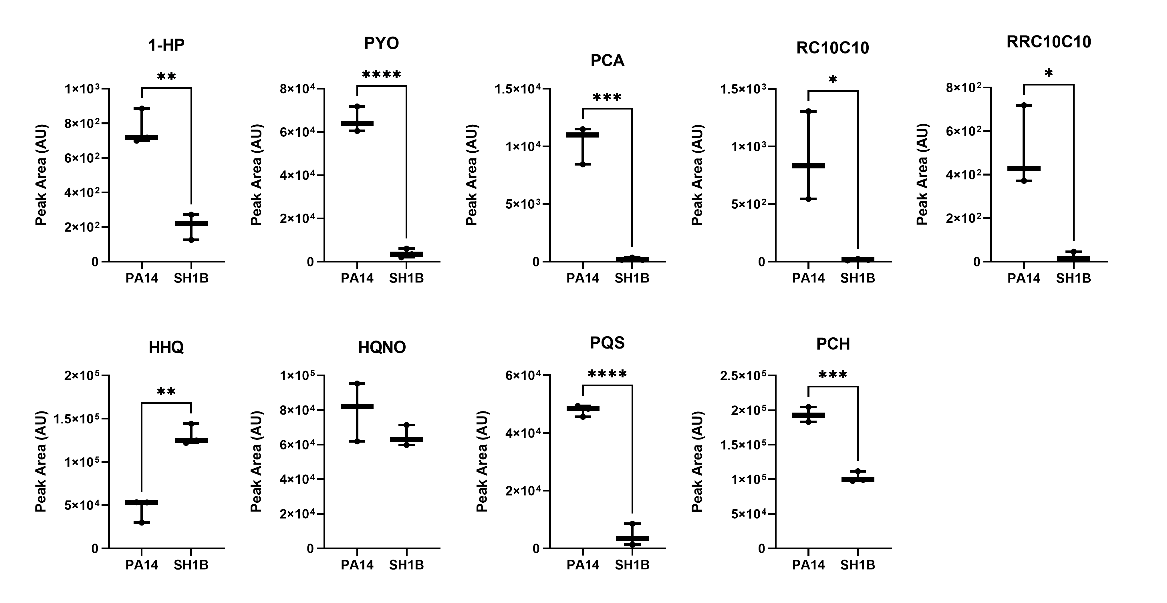


Figure S9. Differential levels of secondary metabolites produced by PA14 and SH1B in SCFM2. PCN was below the limit of quantitation. Box plots represent the 25 to 75th percentiles, with a line at the median. Error bars indicate the minimum to maximum. Individual sample values shown (n = 3 biological replicates per strain). Unpaired two-tailed T-tests. * p < 0.05; ** p < 0.01; *** p < 0.005; **** p < 0.001


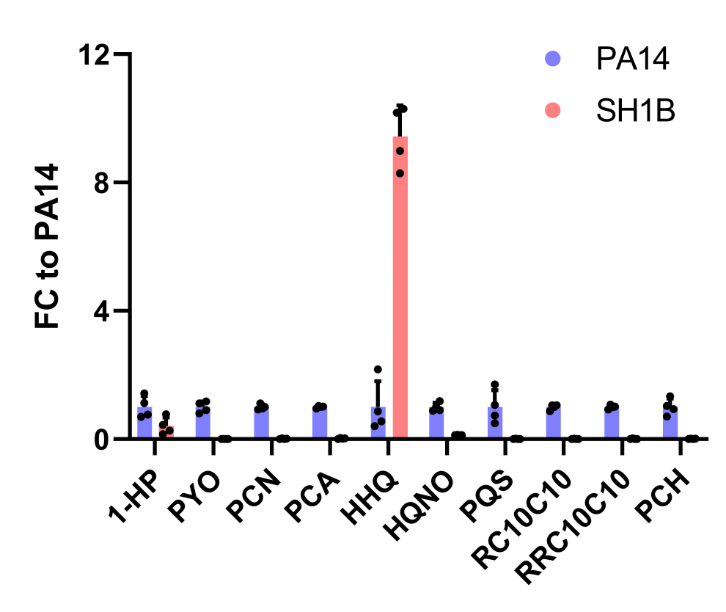


Figure S10. Ratio of secondary metabolite levels produced by SH1B compared to PA14 in LB.


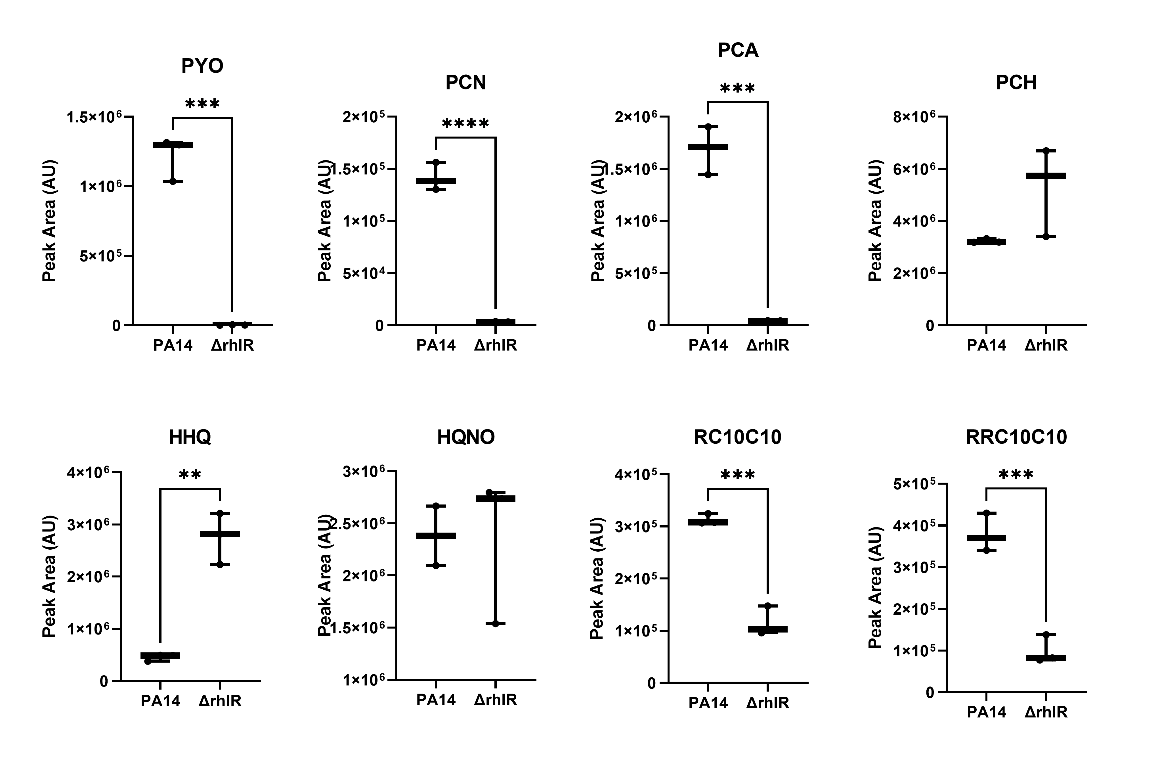


Figure S11. Differential levels of secondary metabolites produced by PA14 and ΔrhlR in LB (MSV000083500). 1‑HP and PQS were below the limit of quantitation. Box plots represent the 25 to 75th percentiles, with a line at the median. Error bars indicate the minimum to maximum. Individual sample values shown (n = 3 biological replicates per strain). Unpaired two-tailed T-tests. * p < 0.05; ** p < 0.01; *** p < 0.005; **** p < 0.001


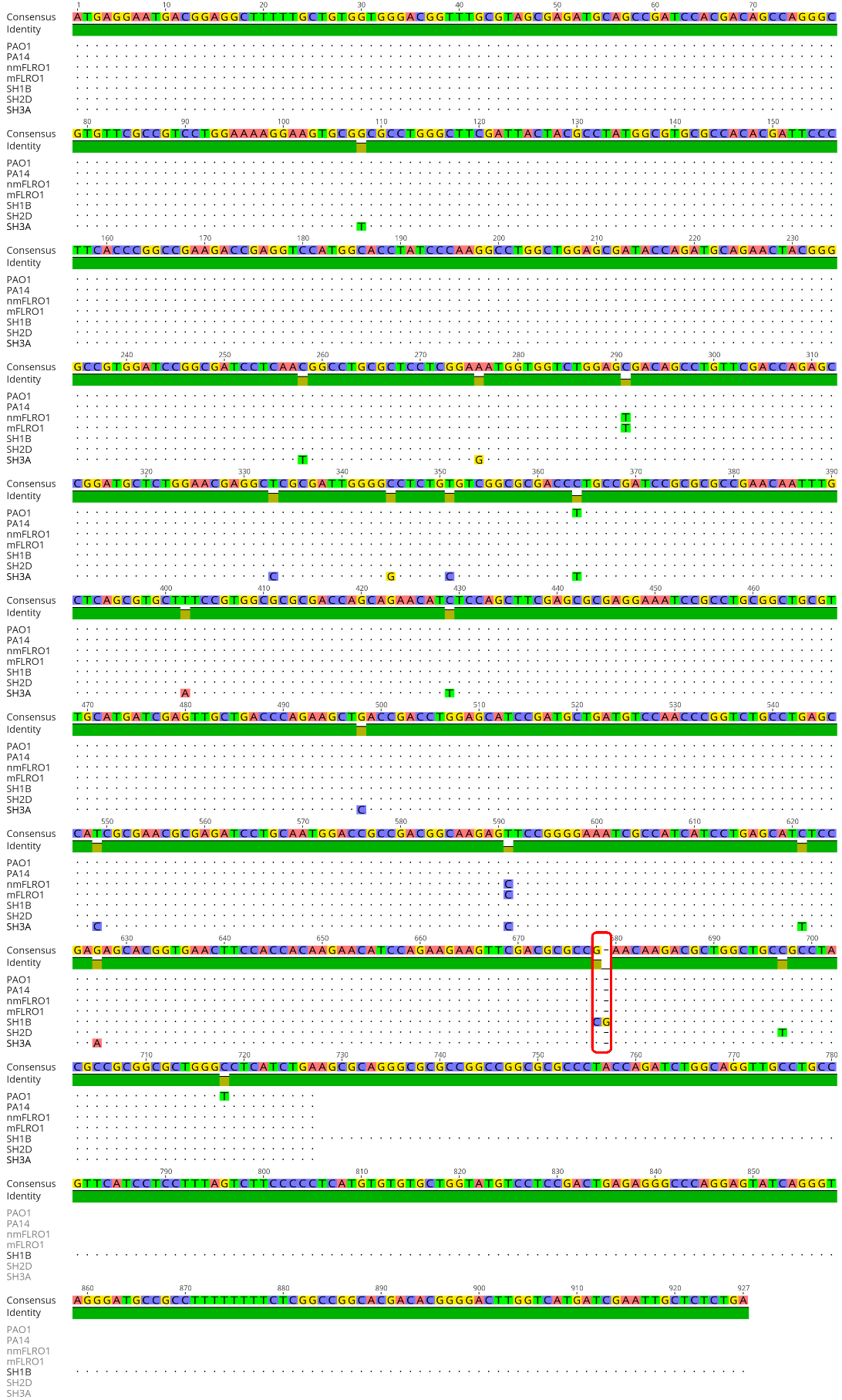


Figure S12. The rhlR gene sequences of PAO1, PA14, nmFLRO1, mFLRO1, SH1B, SH2D, and SH3A, with a red box highlighting single nucleotide insertion at position 678 in SH1B.


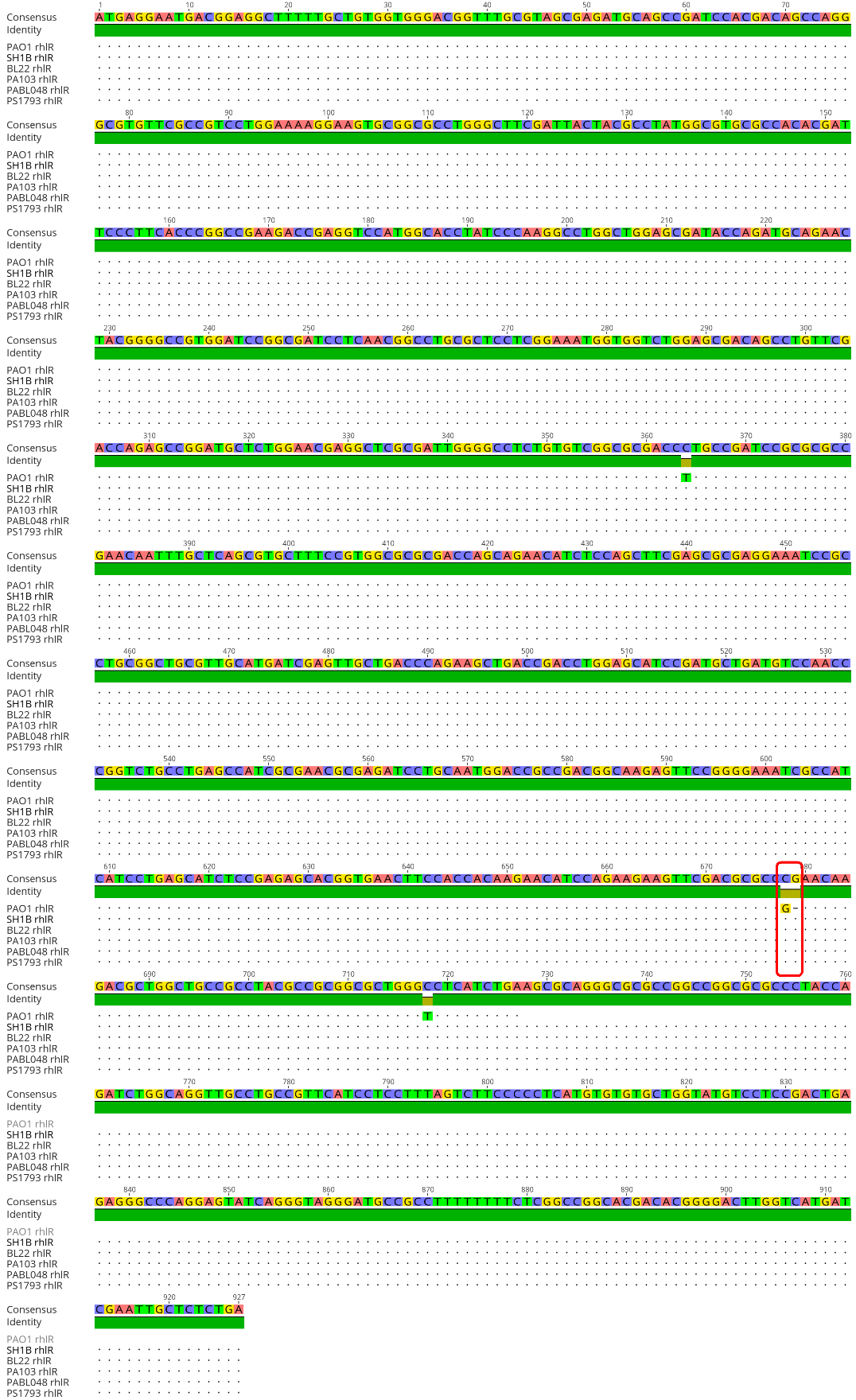


Figure S13. The rhlR gene sequences of PAO1, SH1B, BL22, PA103, PABL048, and PS1793, with a red box highlighting single nucleotide insertion at position 678 in the isolates compared to PAO1.

Table S2. **Secondary Metabolite Annotation**

| **Molecular Family** | **CMN Cluster ID** | **Annotation** | **Adduct** | **Measured m/z** | **RT** | **Calculated m/z** | **Mass Defect (ppm)** | **Annotation Level^#^** |
| --- | --- | --- | --- | --- | --- | --- | --- | --- |
| Phenazine | 462;463;465;466;467;468;469;480;488;512;541 | 1-HP | [M+H] | 197.0718 | 4.2 | 197.0709 | 4.57 | 1 |
| Phenazine | 828;829;831 | PYO | [M+H] | 211.0849 | 0.7 | 211.0866 | -8.05 | 1 |
| Phenazine | 828;829;831 | PYO | [M+H] | 211.0859 | 2.0 | 211.0866 | -3.32 | 1 |
| Phenazine | 1006 | PCN | [M+H] | 224.0822 | 4.0 | 224.0818 | 1.79 | 1 |
| Phenazine | 1018;1019;1020;1021;1022;1035 | PCA | [M+H] | 225.0659 | 4.5 | 225.0659 | 0 | 1 |
| Quinolone | 899;903;910 | C5-HQ | [M+H] | 216.1382 | 4.2 | 216.1383 | -0.46 | 3 |
| Quinolone | 975 | C4-QNO | [M+H] | 218.1180 | 3.8 | 218.1176 | 1.83 | 3 |
| Quinolone | 1236 | C6-HQ | [M+H] | 230.1536 | 4.6 | 230.1539 | -1.3 | 3 |
| Quinolone | 1390 | C5-QNO | [M+H] | 232.1332 | 4.4 | 232.1332 | 0 | 3 |
| Quinolone | 1529 | C7-HQ (HHQ) | [M+H] | 244.1694 | 5.2 | 244.1696 | -0.82 | 1 |
| Quinolone | 1571 | C6-QNO | [M+H] | 246.1488 | 4.8 | 246.1489 | -0.41 | 3 |
| Quinolone | 1830 | C8:1-HQ | [M+H] | 256.1697 | 5.7 | 256.1696 | 0.39 | 3 |
| Quinolone | 1849 | C7:1-QNO | [M+H] | 258.1488 | 5.1 | 258.1489 | -0.39 | 3 |
| Quinolone | 1849 | C8:1-HQ | [M+H] | 258.1851 | 5.7 | 258.1852 | -0.39 | 3 |
| Quinolone | 1861;1857;1864;1856;1858;1859;1862 | C7-QNO (HQNO) | [M+H] | 260.1642 | 5.3 | 260.1645 | -1.15 | 1 |
| Quinolone | 1861;1857;1864;1856;1858;1859;1862 | PQS | [M+H] | 260.1643 | 5.6 | 260.1645 | -0.77 | 1 |
| Quinolone | 1938;1937 | C9:1-HQ | [M+H] | 270.1851 | 6.1 | 270.1852 | -0.37 | 2 |
| Quinolone | 2133 | C8:1-QNO | [M+H] | 272.1643 | 5.5 | 272.1645 | -0.73 | 2 |
| Quinolone | 2138;2135 | C9-HQ (NHQ) | [M+H] | 272.2005 | 6.1 | 272.2009 | -1.47 | 2 |
| Quinolone | 2498 | C8-QNO | [M+H] | 274.1800 | 5.7 | 274.1802 | -0.73 | 2 |
| Quinolone | 2526 | PQS-OH | [M+H] | 276.1596 | 5.9 | 276.1594 | 0.72 | 3 |
| Quinolone | 2668;2670 | C10:1-HQ | [M+H] | 284.2011 | 6.1 | 284.2009 | 0.7 | 3 |
| Quinolone | 2668;2670 | C10:1-HQ | [M+H] | 284.2011 | 6.6 | 284.2009 | 0.7 | 3 |
| Quinolone | 2709;2710;2711;2712 | C9:1-QNO | [M+H] | 286.1799 | 5.9 | 286.1802 | -1.05 | 2 |
| Quinolone | 2709;2710;2711;2712 | C9:1-PQS | [M+H] | 286.1800 | 7.0 | 286.1802 | -0.7 | 3 |
| Quinolone | 2738;2739;2740;2741 | C9-PQS | [M+H] | 288.1956 | 6.5 | 288.1956 | 0 | 2 |
| Quinolone | 2738;2739;2740;2741 | C9-QNO (NQNO) | [M+H] | 288.1956 | 6.1 | 288.1956 | 0 | 3 |
| Quinolone | 2738;2739;2740;2741 | C9-HQ-OH | [M+H] | 288.1957 | 5.0 | 288.1956 | 0.35 | 3 |
| Quinolone | 2840 | C11:2-HQ | [M+H] | 296.2010 | 6.6 | 296.2009 | 0.34 | 3 |
| Quinolone | 2896 | C11:1-HQ | [M+H] | 298.2162 | 6.5 | 298.2165 | -1.01 | 2 |
| Quinolone | 2943 | C10:1-QNO | [M+H] | 300.1961 | 6.2 | 300.1958 | 1 | 2 |
| Quinolone | 2943 | C11-HQ | [M+H] | 300.2318 | 7.0 | 300.2322 | -1.33 | 2 |
| Quinolone | 3051;3053 | C9-QNO-OH | [M+H] | 304.1908 | 5.2 | 304.1907 | 0.33 | 3 |
| Quinolone | 3051;3053 | C9-PQS-OH | [M+H] | 304.1909 | 6.6 | 304.1907 | 0.66 | 3 |
| Quinolone | 3153;3176 | C11:2-QNO/C11:1-PQS | [M+H] | 312.1956 | 6.4 | 312.1958 | -0.64 | 3 |
| Quinolone | 3153;3176 | C12:1-HQ | [M+H] | 312.2320 | 6.9 | 312.2322 | -0.64 | 3 |
| Quinolone | 3207;3210 | C11:1-QNO | [M+H] | 314.2110 | 6.5 | 314.2115 | -1.59 | 3 |
| Quinolone | 3207;3210 | C11:1-PQS | [M+H] | 314.2114 | 6.8 | 314.2115 | -0.32 | 3 |
| Quinolone | 3230 | C11-QNO | [M+H] | 316.2268 | 7.0 | 316.2271 | -0.95 | 3 |
| Quinolone | 3283 | C13:2-HQ | [M+H] | 324.2316 | 7.3 | 324.2322 | -1.85 | 3 |
| Quinolone | 3321 | C13:1-HQ | [M+H] | 326.2475 | 7.3 | 326.2479 | -1.23 | 3 |
| Quinolone | 3409;3414 | C12:1-QNO/C12:1-PQS | [M+H] | 328.2273 | 6.9 | 328.2271 | 0.61 | 3 |
| Quinolone | 3517 | C13:2-QNO/C13:2-PQS | [M+H] | 340.2271 | 7.0 | 340.2271 | 0 | 3 |
| Quinolone | 3554 | C13:1-QNO | [M+H] | 342.2430 | 7.2 | 342.2428 | 0.58 | 3 |
| Quinolone | 3571 | C15:1-HQ | [M+H] | 354.2785 | 8.1 | 354.2791 | -1.69 | 3 |
| Quinolone | 3762 | C15-HQ | [M+H] | 356.2950 | 8.8 | 356.2948 | 0.56 | 3 |
| Quinolone | 4750 | C17:1-HQ | [M+H] | 382.3098 | 8.9 | 382.3104 | -1.57 | 3 |
| Siderophore | 3284 | PCH | [M+H] | 325.0673 | 4.6 | 325.0675 | -0.62 | 2 |
| Siderophore | 3284 | PCH | [M+H] | 325.0672 | 5.0 | 325.0675 | -0.92 | 2 |
| Rhamnolipid | 5930 | RC8C10 | [M+Na] | 499.2882 | 6.6 | 499.2878 | 0.8 | 1 |
| Rhamnolipid | 6052 | RC10C10 | [M+Na] | 527.3186 | 7.2 | 527.3191 | -0.95 | 1 |
| Rhamnolipid | 6269 | RC10C11 | [M+Na] | 541.3336 | 7.6 | 541.3347 | -2.03 | 1 |
| Rhamnolipid | 6806 | RC10C12:1 | [M+Na] | 553.3348 | 7.7 | 553.3347 | 0.18 | 1 |
| Rhamnolipid | 6812 | RC10C12 | [M+Na] | 555.3498 | 8.0 | 555.3504 | -1.08 | 1 |
| Rhamnolipid | 6878 | R(OAc)C10C10 | [M+Na] | 569.3300 | 7.8 | 569.3290 | 1.76 | 3 |
| Rhamnolipid | 6898 | RC12C12:1 | [M+Na] | 581.3648 | 8.5 | 581.3660 | -2.06 | 1 |
| Rhamnolipid | 6900 | RC12C12 | [M+Na] | 583.3820 | 8.8 | 583.3817 | 0.51 | 1 |
| Rhamnolipid | 8214 | RRC10C10 | [M+Na] | 673.3772 | 6.7 | 673.3770 | 0.3 | 1 |
| Rhamnolipid | 8367 | RRC10C12:1 | [M+Na] | 699.3922 | 7.2 | 699.3926 | -0.57 | 1 |
| Rhamnolipid | 8372 | RRC10C12 | [M+Na] | 701.4073 | 7.4 | 701.4083 | -1.43 | 1 |
| Rhamnolipid | 8377 | R(OAc)RC10C10 | [M+Na] | 715.3870 | 7.3 | 715.3875 | -0.7 | 3 |
| Rhamnolipid | 8395 | RRC12C12 | [M+Na] | 729.4410 | 8.2 | 729.4396 | 1.92 | 1 |
| Acyl Putrescine | 2862 | Putrescine C14:1 | [M+H] | 297.2898 | 5.3 | 297.2900 | -0.67 | 3 |
| Acyl Putrescine | 2935 | Putrescine C14:0 | [M+H] | 299.3061 | 5.7 | 299.3057 | 1.34 | 3 |
| Acyl Putrescine | 3206 | Putrescine C15:0 | [M+H] | 313.3217 | 5.8 | 313.3213 | 1.28 | 3 |
| Acyl Putrescine | 3282 | Putrescine C16:2 | [M+H] | 323.3060 | 5.7 | 323.3057 | 0.93 | 3 |
| Acyl Putrescine | 3314 | Putrescine C16:1 | [M+H] | 325.3216 | 5.8 | 325.3213 | 0.92 | 2 |
| Acyl Putrescine | 3388 | Putrescine C16:0 | [M+H] | 327.3373 | 6.2 | 327.3370 | 0.92 | 3 |
| Acyl Putrescine | 3546 | Putrescine C17:1 | [M+H] | 339.3374 | 6.0 | 339.3370 | 1.18 | 3 |
| Acyl Putrescine | 3661 | Putrescine C18:2 | [M+H] | 351.3376 | 6.2 | 351.3370 | 1.71 | 3 |
| Acyl Putrescine | 3705 | Putrescine C18:1 | [M+H] | 353.3529 | 6.3 | 353.3526 | 0.85 | 2 |
| Acyl Putrescine | 3945 | Putrescine C19:1 | [M+H] | 367.3690 | 6.4 | 367.3683 | 1.91 | 3 |

REFERENCES

1. Jacobs MA, Alwood A, Thaipisuttikul I, Spencer D, Haugen E, Ernst S, Will O, Kaul R, Raymond C, Levy R, Chun-Rong L, Guenthner D, Bovee D, Olson MV, Manoil C. 2003. Comprehensive transposon mutant library of Pseudomonas aeruginosa. Proc Natl Acad Sci U S A 100:14339-44.

2. Lee DG, Urbach JM, Wu G, Liberati NT, Feinbaum RL, Miyata S, Diggins LT, He J, Saucier M, Deziel E, Friedman L, Li L, Grills G, Montgomery K, Kucherlapati R, Rahme LG, Ausubel FM. 2006. Genomic analysis reveals that Pseudomonas aeruginosa virulence is combinatorial. Genome Biol 7:R90.

3. Quinn RA, Whiteson K, Lim YW, Salamon P, Bailey B, Mienardi S, Sanchez SE, Blake D, Conrad D, Rohwer F. 2015. A Winogradsky-based culture system shows an association between microbial fermentation and cystic fibrosis exacerbation. ISME J 9:1024-38.

4. Geisenberger O, Givskov M, Riedel K, Hoiby N, Tummler B, Eberl L. 2000. Production of N-acyl-L-homoserine lactones by P. aeruginosa isolates from chronic lung infections associated with cystic fibrosis. FEMS Microbiol Lett 184:273-8.
